# Continuous prolonged prone positioning in COVID-19-related ARDS: a multicenter cohort study from Chile

**DOI:** 10.1186/s13613-022-01082-w

**Published:** 2022-11-28

**Authors:** Rodrigo A. Cornejo, Jorge Montoya, Abraham I. J. Gajardo, Jerónimo Graf, Leyla Alegría, Romyna Baghetti, Anita Irarrázaval, César Santis, Nicolás Pavez, Sofía Leighton, Vinko Tomicic, Daniel Morales, Carolina Ruiz, Pablo Navarrete, Patricio Vargas, Roberto Gálvez, Victoria Espinosa, Marioli Lazo, Rodrigo A. Pérez-Araos, Osvaldo Garay, Patrick Sepúlveda, Edgardo Martinez, Alejandro Bruhn, the SOCHIMI Prone-COVID-19 Group, Nicole Rossel, María José Martin, Juan Nicolás Medel, Vanessa Oviedo, Magdalena Vera, Vicente Torres, José Miguel Montes, Álvaro Salazar, Carla Muñoz, Francisca Tala, Mariana Migueles, Claudia Ortiz, Felipe Gómez, Luis Contreras, Itzia Daviu, Yurimar Rodriguez, Carol Ortiz, Andrés Aquevedo, Rodrigo Parada, Cristián Vargas, Miguel Gatica, Dalia Guerrero, Araceli Valenzuela, Diego Torrejón

**Affiliations:** 1 Unidad de Pacientes Críticos, Departamento de Medicina, Hospital Clínico Universidad de Chile, Dr. Carlos Lorca Tobar 999, 2º Piso, Independencia, Santiago, Chile; 2 Departamento de Paciente Crítico, Clínica Alemana de Santiago, Santiago, Chile; 3 Facultad de Medicina, Clínica Alemana-Universidad de Desarrollo, Santiago, Chile; 4 Departamento de Medicina Intensiva, Facultad de Medicina, Edificio Académico Escuela de Medicina, Pontificia Universidad Católica de Chile, Diagonal Paraguay 362, 6º Piso, Santiago, Chile; 5 Unidad de Pacientes Críticos Adultos, Hospital Carlos Van Buren, Valparaíso, Chile; 6 Unidad de Paciente Crítico, Hospital La Serena, Coquimbo, Chile; 7 Unidad de Pacientes Críticos, Hospital Barros Luco Trudeau, Santiago, Chile; 8 Departamento de Medicina Interna Campus Sur, Universidad de Chile, Santiago, Chile; 9 Unidad de Pacientes Críticos, Hospital Regional de Concepción, Concepción, Chile; 10 Departamento de Medicina Interna, Facultad de Medicina, Universidad de Concepción, Concepción, Chile; 11 Unidad de Pacientes Críticos, Hospital Padre Hurtado, Santiago, Chile; 12 Unidad de Paciente Crítico, Hospital Clinico Regional de Antofagasta, Antofagasta, Chile; 13 Facultad de Medicina y Odontología, Universidad de Antofagasta, Antofagasta, Chile; 14 Unidad de Pacientes Críticos, Hospital Clínico Dra. Eloisa Diaz I-La Florida, Santiago, Chile; 15 Unidad de Paciente Crítico, Complejo Asistencial Dr. Sótero del Río, Santiago, Chile; 16 Unidad de Pacientes Críticos, Hospital Clínico Herminda Martín, Chillán, Chile; 17 Unidad de Paciente Crítico, Hospital de Urgencia Asistencia Pública, Santiago, Chile; 18 Facultad de Medicina, Universidad Finis Terrae, Santiago, Chile; 19 Unidad de Pacientes Críticos, Hospital Regional de Iquique, Iquique, Chile; 20 Unidad de Paciente Crítico, Clínica Alemana de Temuco, Temuco, Chile; 21 Facultad de Medicina, Universidad de La Frontera, Temuco, Chile; 22 Center of Acute Respiratory Critical Illness (ARCI), Santiago, Chile

**Keywords:** Acute respiratory distress syndrome, Mechanical ventilation, Prone positioning, Coronavirus disease 2019

## Abstract

**Background:**

Prone positioning is currently applied in time-limited daily sessions up to 24 h which determines that most patients require several sessions. Although longer prone sessions have been reported, there is scarce evidence about the feasibility and safety of such approach. We analyzed feasibility and safety of a continuous prolonged prone positioning strategy implemented nationwide, in a large cohort of COVID-19 patients in Chile.

**Methods:**

Retrospective cohort study of mechanically ventilated COVID-19 patients with moderate-to-severe acute respiratory distress syndrome (ARDS), conducted in 15 Intensive Care Units, which adhered to a national protocol of continuous prone sessions  ≥ 48 h and until PaO_2_:FiO_2_ increased above 200 mm Hg. The number and extension of prone sessions were registered, along with relevant physiologic data and adverse events related to prone positioning. The cohort was stratified according to the first prone session duration: Group A, 2–3 days; Group B, 4–5 days; and Group C, > 5 days. Multivariable regression analyses were performed to assess whether the duration of prone sessions could impact safety.

**Results:**

We included 417 patients who required a first prone session of 4 (3–5) days, of whom 318 (76.3%) received only one session. During the first prone session the main adverse event was grade 1–2 pressure sores in 97 (23.9%) patients; severe adverse events were infrequent with 17 non-scheduled extubations (4.2%). 90-day mortality was 36.2%. Ninety-eight patients (24%) were classified as group C; they exhibited a more severe ARDS at baseline, as reflected by lower PaO_2_:FiO_2_ ratio and higher ventilatory ratio, and had a higher rate of pressure sores (44%) and higher 90-day mortality (48%). However, after adjustment for severity and several relevant confounders, prone session duration was not associated with mortality or pressure sores.

**Conclusions:**

Nationwide implementation of a continuous prolonged prone positioning strategy for COVID-19 ARDS patients was feasible. Minor pressure sores were frequent but within the ranges previously described, while severe adverse events were infrequent. The duration of prone session did not have an adverse effect on safety.

**Supplementary Information:**

The online version contains supplementary material available at 10.1186/s13613-022-01082-w.

## Background

Although prone positioning has been shown to decrease mortality in mechanically ventilated patients with moderate-to-severe acute respiratory distress syndrome (ARDS), it is still underused worldwide [1, 2]. One of the reasons to avoid using prone positioning is the increased workload associated with daily repositioning patients [1].

The strategy of applying time-limited prone sessions can be traced back to the first reports in the seventies, when it was used for short time periods in severely hypoxemic ARDS patients [3]. The first randomized controlled trials of prone positioning in ARDS maintained this approach applying daily 6–8 h sessions [4]. After the negative results of these first trials and based on physiologic studies indicating that oxygenation continued improving beyond 8 h [5], subsequent clinical trials applied longer sessions [1, 6, 7]. After the release of the PROSEVA trial, the first study to clearly show a survival advantage of prone positioning [1], daily sessions of 16–20 h became the standard still applied today.

However, most patients require several prone sessions before a stable improvement in gas exchange can be reached [1] and the rationale for returning patients back to supine position once a day remains unclear. Furthermore, daily shifts to supine position have their own complexities. First, it increases staff workload; this factor became critical during the COVID-19 pandemic [8–13]. Second, oxygenation may rapidly deteriorate after returning to supine, particularly in the first days [1, 8, 14]. Third, some feared adverse events related to prone positioning may occur during patient repositioning [15]. Fourth, as the benefits of prone positioning are most likely due to a more homogenous distribution of lung strain, lung protection may be compromised while turned back to supine position [16, 17].

Prolonged sessions of prone positioning beyond 24 h have been reported mainly in small series [18–20]. In 2005, some centers in Chile began to apply prolonged prone positioning in continuous sessions, without a time limit, but extended until reaching a predefined oxygenation threshold while in prone position [18]. When the COVID-19 pandemic spread to Chile, anticipating rapid expansion in ICU capacity and staff overload, national experts recommended the routine use of this strategy for mechanically ventilated patients with moderate-to-severe ARDS, which was adopted by most centers along the country. We thought that analyzing this large and unique experience could contribute to define whether continuous prolonged prone positioning may become an alternative to the current approach of intermittent daily prone positioning.

The goal of the present study was to describe the feasibility and safety of a strategy of continuous prolonged prone positioning applied routinely in mechanically ventilated patients, on a nationwide scale, during the first wave of the COVID-19 pandemic. In addition, we sought to determine whether the duration of the first prone session could impact safety.

## Methods

### Study design

We performed a multicenter, historical cohort study in mechanically ventilated patients with COVID-19-related ARDS who required prone positioning, aimed to analyze the feasibility and safety of a continuous prone positioning strategy. A representative sample of 30 ICUs (public, university and private institutions) were invited through the Chilean Society of Intensive Care Medicine (SOCHIMI). We recruited physicians from each participating ICU as lead site investigators. Each ICU provided data concerning its resources and prone positioning protocol, before and during the first wave of COVID-19 pandemic (Additional file 1: Table S2). The enrollment window consisted of 8 consecutive weeks, as selected by each ICU within the period corresponding to the first wave of COVID-19 (April 1st to August 31st, 2020). A standard form was used to collect the data from clinical files. The study was approved by an Institutional Review Board which waived informed consent (N° 063/2020, Hospital Clínico Universidad de Chile).

### Strategy of continuous prolonged prone position

The national recommendations for prone positioning aimed to facilitate its use without overloading staff with continuous shifts between supine and prone position. Prone positioning was indicated in mechanically ventilated patients with moderate-to-severe COVID-19-related-ARDS with a PaO_2_:FiO_2_ ratio below 150 mm Hg after optimizing protective mechanical ventilation settings. Low tidal volume and moderate PEEP levels, but not a specific PEEP titration strategy, were recommended in both supine and prone positions (Additional file 1: Fig. S1). Considering that the large majority of ARDS patients require prone positioning for more than 2 days [12], we defined that prone sessions should last at least 48 h, and that they should extend until PaO_2_:FiO_2_ was above 200 mm Hg while in prone position. This threshold was based on the notion that oxygenation frequently decreases after turning patients to supine and our goal was to minimize the chance that the patient could require a second session.

### Patients, study design, and data collection

Mechanically ventilated patients with COVID-19-related ARDS, who received at least one session of prone positioning  > 48 h, within the 8-week enrollment window, were recruited. A positive polymerase chain reaction test for SARS-CoV-2 was required for COVID-19 diagnosis, and ARDS was defined according to Berlin criteria [11]. Exclusion criteria were age younger than 18 years and patients with missing relevant data.

Trained personnel registered patients’ demographic characteristics, severity scores and respiratory support at hospital and ICU admission. Respiratory and hemodynamic parameters were collected before/after intubation, before/during prone sessions, and after return to supine position. Sedation before/during prone session was also registered. Ventilatory ratio was calculated to estimate pulmonary dead space [21]. The time course of PaO_2_:FiO_2_ ratio, static compliance of the respiratory system (static compliance), and ventilatory ratio was analyzed. Different conditions for prematurely interrupting prone sessions, based on the PROSEVA trial criteria, were registered (Additional file 1: Table S8). Day 1 was defined as the first day in prone positioning.

As the duration of prone sessions was very heterogeneous, in order to better analyze the potential influence of this variable on safety, and considering previous references [18, 19, 22], we divided the cohort in three groups according to the duration of their first prone session: group A, 2–3 days; group B, 4–5 days; and group C, > 5 days.

### Safety and outcomes

The incidences of adverse events potentially related to the first prone positioning session were specified: pressure sores in ventral surfaces (from head, chest, abdomen, and groin) staged according to the National Pressure Ulcer Advisory Panel’s updated pressure ulcer staging system (NPUAP) [23], displacement of vascular catheters, non-scheduled extubation, and endotracheal tube obstruction. The cumulative incidence of pressure sores up to day 7 was also assessed. Regarding outcomes, we recorded 90-day mortality, ICU and hospital mortality, duration of mechanical ventilation, tracheostomy requirements and hospital/ICU length of stay.

For missing data, the last time with available data was used and considered as in-risk time.

Prior to analysis, all data were screened for potentially erroneous data, and verified or corrected by site investigators. This study was conducted in accordance with the STROBE guideline [24].

### Statistics

The Shapiro–Wilk test was used to assess data normality. Descriptive statistics were reported as median (IQR [interquartile range p25-p75]) or count (%). Data before and after intubation and before and after prone positioning onset were compared by Wilcoxon signed-rank test. Comparisons among subgroups were performed by Kruskal–Wallis or Fisher exact tests. The evolution of static compliance, PaO_2_:FiO_2_ ratio, and ventilatory ratio was analyzed through mixed effects regression models, considering each patient as a random effect, and relevant time points as fixed effect. Multivariable regression analyses were performed to assess whether the duration of prone sessions influenced 90-day mortality and the risk of pressure sores. To control the association of the duration of the first prone session with 90-day mortality, we identified variables associated to the exposure (i.e., groups according to prone session duration) by ordered logistic regressions (Additional file 1: Table S9) and variables associated to the outcome (Additional file 1: Table S10). A directed acyclic graph was used to select the confounding factors (Additional file 1: Fig. S5) to control for: SOFA score at baseline, vasoactive support at day 1 in prone and respiratory variables (static compliance, PaO_2_:FiO_2_ and ventilatory ratio) at day 2 in prone [9, 10, 12, 25]. In the case of cumulative incidence of pressure sores up to day 7, we included the groups according to the duration of their first prone session and SOFA score [26–29] in the logistic regressions.

Variables with missing data were reported (Additional file 1: Table S3), assumed to be missing at random, and an available-case analysis was performed. Observed characteristics between patients with complete and incomplete data were compared (Additional file 1: Table S4). Analyses were performed in Stata v 14.0 (StataCorp) and graphs plotted in GraphPad Prism v 8.0.

## Results

### Participating centers and enrolled patients

Fifteen centers accepted to participate in the study: 11 public, 2 private and 2 from university hospitals. All participating centers implemented the national recommendations for prone positioning and performed a protocol that included a checklist, which was systematically applied in 14 (87%) centers (Additional file 1: Fig. S1, Table S2).

Of 2822 mechanically ventilated patients admitted to the participating centers between April 1st to August 31st, 2020, 1795 (63.6%) were treated with prone positioning (Additional file 1: Table S1). During the 8-week enrollment window selected by each center, 547 patients were included in the database, but after excluding 130 patients due to incomplete data, 417 were finally analyzed (Additional file 1: Fig. S2). Patient characteristics are outlined in Table 1.

**Table 1:** Clinical characteristics at baseline according to the duration of the first prone session

	Total (*n* = 417)	Group A (*n* = 191)	Group B (*n* = 128)	Group C (*n* = 98)	*p*-value
Age, years, median (IQR)	62 (52–68)	61 (52–68)	61 (47–67)	63 (54–69)	0.188
Sex, *n* (%)					
Male	301 (72.2)	135 (70.7)	95 (74.2)	71 (72.4)	0.796
Female	116 (27.8)	56 (29.3)	33 (25.8)	27 (27.6)	0.796
Body mass index (kg/m^2^), median (IQR)	30 (27–34)	29 (26–33)	30 (26–34)	31 (27–35)	0.098
Comorbidities, *n* (%)					
Hypertension	232 (55.6)	108 (56.5)	70 (54.7)	54 (55.1)	0.951
Type 2 diabetes mellitus	166 (39.8)	85 (44.5)	43 (33.6)	38 (38.8)	0.148
Coronary heart disease	17 (4.1)	6 (3.1)	3 (2.3)	8 (8.2)	0.074
Chronic liver disease	10 (2.4)	5 (2.6)	4 (3.1)	1 (1)	0.652
Immunosuppression	6 (1.4)	5 (2.6)	–	1 (1)	0.144
Chronic kidney disease	20 (4.8)	13 (6.8)	3 (2.3)	4 (4.1)	0.182
Obesity	147 (35.3)	61 (31.9)	47 (36.7)	39 (39.8)	0.368
Other	121 (29)	58 (30.4)	35 (27.3)	28 (28.6)	0.842
None	63 (15.1)	32 (16.8)	18 (14.1)	13 (13.3)	0.712
APACHE II score, median (IQR)	14 (10–18)	14 (10–18)	14 (10–18)	13 (10–17)	0.748
SOFA score, median (IQR)	5 (4–7)	5 (4–8)	5 (4–7)	4 (4–6)	0.246
Sepsis, *n* (%)	100 (24.3)	37 (19.7)	29 (22.8)	34 (35.1)	0.017
Time between (days), median (IQR)					
Diagnosis to hospital admission	0 (0–3)	0 (0–3)	0 (0–4)	0 (0–1)	0.096
Hospital to ICU admission	1 (0–3)	1 (0–3)	1 (0–3)	1 (0–4)	0.086
Diagnosis to invasive ventilation	3 (1–6)	3 (0–6)	3 (1–7)	3 (1–5)	0.940
Invasive ventilation to prone position	1 (0–1)	1 (0–2)	1 (0–2)	0 (0–1)	< 0.001

Group A patients remained 2–3 days in prone position during their first session. Group B patients remained 4–5 days in prone position during their first session. Group C patients remained more than 5 days in prone position during their first session

*APACHE II* Acute Physiology and Chronic Health Disease Classification System II, *SOFA* Sepsis Related Organ Failure Assessment, *Diagnosis* COVID-19 diagnosis, *ICU* intensive care unit

Before intubation, almost half of patients were treated with high flow nasal cannula (HFNC) and 29% with awake prone (Additional file 1: Table S5). Once intubated, patients were ventilated with tidal volume 6.2 (5.7–6.8) mL/Kg ideal body weight (IBW) and PaO_2_:FiO_2_ ratio before starting prone positioning was 119 (85–153) mm Hg (Table 2).

**Table 2:** Respiratory parameters before and after starting the first prone session

	Total (*n* = 417)	Group A (*n* = 191)	Group B (*n* = 128)	Group C (*n* = 98)	*p* value
Respiratory rate, breaths per minute, median (IQR)					
* Before starting prone positioning*	26 (23–30)	25 (22–28)	26 (24–30)	28 (24–30)	< 0.001
* At day 1 in prone positioning*	26 (24–30)	26 (24–28)	27 (24–30)	28 (26–30)	< 0.001
* Δ Respiratory rate [PP day 1–before PP]*	0 (0–2)	0 (0–2)	0 (0–2)	0 (0–4)	0.260
Tidal volume, mL/kg IBW, median (IQR)					
* Before starting prone positioning*	6.2 (5.7–6.8)	6.3 (5.7–6.9)	6.1 (5.7–6.9)	6.1 (5.7–6.8)	0.530
* At day 1 in prone positioning*	6.2 (5.7–6.8)	6.2 (5.7–6.8)	6.2 (5.7–6.9)	6.2 (5.7–6.8)	0.999
* Δ VT [PP day 1–before PP]*	0.0 (− 0.2, 0.2)	0.0 (− 0.2, 0.1)	0.0 (− 0.2, 0.2)	0.0 (− 0.2, 0.3)	0.235
Plateau pressure, cm H_2_O, median (IQR)					
* Before starting prone positioning*	23 (21–26)	23 (21–25)	24 (21–28)	25 (21–27)	0.003
* At day 1 in prone positioning*	22 (21–25)	22 (20–24)	23 (21–26)	23 (21–25)	0.002
* Δ Plateau pressure [PP day 1–before PP]*	− 1 (−3 to 1)	− 1 (− 3 to 1)	− 1 (− 3 to 1)	− 1 (− 3 to 2)	0.685
Driving pressure, cm H_2_O, median (IQR)					
* Before starting prone positioning*	12 (10–15)	12 (10–14)	13 (11–15)	13 (11–16)	0.005
* At day 1 in prone positioning*	12 (10–14)	11 (10–13)	12 (10–15)	13 (11–14)	< 0.001
* Δ Driving pressure [PP day 1–before PP]*	− 1 (− 2 to 1)	− 1 (− 2 to 1)	0 (− 2 to 1)	− 1 (− 2 to 1)	0.976
PEEP, cm H_2_O, median (IQR)					
* Before starting prone positioning*	10 (8–12)	10 (8–12)	10 (9–12)	10 (8–12)	0.309
* At day 1 in prone positioning*	10 (8–12)	10 (8–12)	10 (8–12)	10 (8–12)	0.686
* Δ PEEP [PP day 1–before PP]*	0 (− 2 to 0)	0 (−1 to 0)	0 (−2 to 0)	0 (−1 to 0)	0.385
Static compliance, mL/cm H_2_O, median (IQR)					
* Before starting prone positioning*	31 (25–38)	32 (27–38)	30 (25–38)	30 (23–36)	0.133
* At day 1 in prone positioning*	32 (27–39)	33 (28–40)	32 (26–39)	30 (27–36)	0.007
* Δ Static compliance [PP day 1–before PP]*	2 (− 3 to 5)	2 (− 2 to 6)	1 (− 3 to 4)	1 (− 4 to 4)	0.070
PaO_2_, mm Hg, median (IQR)					
* Before starting prone positioning*	75 (64–87)	76 (66–87)	71 (63–83)	77 (62–89)	0.168
* At day 1 in prone positioning*	84 (72–102)	89 (75–109)	82 (71–96)	80 (69–97)	0.013
* Δ PaO*_*2*_* [PP day 1–before PP]*	10 (− 3 to 28)	12 (− 4 to 35)	8 (− 3 to 26)	9 (− 4 to 21)	0.218
FiO_2_, median (IQR)					
* Before starting prone positioning*	0.65 (0.50–0.90)	0.60 (0.50–0.80)	0.65 (0.50–1.0)	0.75 (0.60–1.0)	0.002
* At day 1 in prone positioning*	0.50 (0.40–0.60)	0.40 (0.35–0.50)	0.50 (0.40–0.60)	0.55 (0.50–0.70)	< 0.001
* Δ FiO*_*2*_* [PP day 1–before PP]*	− 0.15 (− 0.3 to − 0.05)	− 0.20 (− 0.35 to − 0.05)	− 0.15 (− 0.35 to 0.0)	− 0.20 (− 0.30 to 0.0)	0.293
PaO_2_:FiO_2_ ratio, median (IQR)					
* Before starting prone positioning*	119 (85–154)	127 (98–162)	114 (86–150)	106 (81–140)	0.003
* At day 1 in prone positioning*	184 (139–240)	224 (174–270)	176 (133–214)	145 (117–183)	< 0.001
* Δ PaO*_*2*_*:FiO*_*2*_* ratio [PP day 1–before PP]*	61 (23–115)	88 (34–151)	53 (19–89)	39 (18–71)	< 0.001
PaCO_2_, mm Hg, median (IQR)					
* Before starting prone positioning*	45 (39–53)	43 (38–48)	47 (40–56)	47 (40–56)	< 0.001
* At day 1 in prone positioning*	45 (40–51)	43 (39–50)	46 (41–54)	47 (43–53)	< 0.001
* Δ PaCO*_*2*_* [PP day 1–before PP]*	0 (− 6 to 6)	0 (− 5 to 6)	− 1 (− 8 to 6)	− 0 (− 6 to 4)	0.408
pH, median (IQR)					
* Before starting prone positioning*	7.34 (7.27–7.40)	7.36 (7.30–7.41)	7.34 (7.26–7.40)	7.31 (7.26–7.36)	< 0.001
* At day 1 in prone positioning*	7.35 (7.29–7.40)	7.37 (7.30–7.41)	7.35 (7.29–7.40)	7.32 (7.28–7.36)	0.004
* Δ pH [PP day 1–before PP]*	0.0 (− 0.0, 0.1)	0.0 (− 0.0, 0.1)	0.0 (− 0.0, 0.1)	0.0 (− 0.0, 0.1)	0.196
Ventilatory ratio, median (IQR)					
* Before starting prone positioning*	1.9 (1.6–2.4)	1.8 (1.5–2.2)	2.1 (1.6–2.7)	2.1 (1.8–2.6)	< 0.001
* At day 1 in prone positioning*	2.0 (1.6–2.5)	1.8 (1.5–2.3)	2.1 (1.7–2.6)	2.2 (1.9–2.8)	< 0.001
* Δ Ventilatory ratio [PP day 1–before PP]*	0.0 (− 0.2 to 0.3)	0.0 (− 0.2 to 0.3)	− 0.0 (− 0.3 to 0.3)	0.0 (− 0.1 to 0.3)	0.441

Group A patients remained 2–3 days in prone position during their first session. Group B patients remained 4–5 days in prone position during their first session. Group C patients remained more than 5 days in prone position during their first session. The data reported as before starting prone positioning (upper half of the table) correspond to the last data registered in the clinical files before turning the patient to prone position for the first time, while the data reported as after starting prone positioning (lower half of the table) correspond to the first data registered in the clinical files collected while the patient was in prone position

*PaO2* partial pressure of arterial oxygen, *FiO2* Inspired oxygen fraction, *PaO2:FiO2* ratio: ratio of partial pressure of arterial oxygen to inspired oxygen fraction, *PaCO*2 partial pressure of arterial carbon dioxide, *PEEP* Positive end-expiratory pressure, Static compliance: Static respiratory system compliance, Ventilatory ratio is a unit less index calculated as (minute ventilation in ml/min x PaCO2)/(Ideal body weight × 100 × 37.5)

### Prone positioning sessions

Prone positioning was initiated early after intubation (1 [0–1] days). Most patients (76.3%) required only one prone session, with a median duration of 4 (3–5) days; 21.1% required two sessions and 2.7% a third session (Additional file 1: Table S7). In 113 (27%) patients the first prone session was interrupted prematurely, either due to life-threatening conditions in 47 (11.3%) patients, or just for a clinical decision of the attending physician in 66 (15.8%) patients (Additional file 1: Table S8). For patients who required a second prone session, time elapsed in supine position between prone sessions was 2 (1–5) days. The cumulative time in prone positioning was 4 (3–7) days, with longer times in patients with repeated sessions (Table 3). The percentage of patients using neuromuscular blockade significantly increased with the change to prone; meanwhile, a mild increase in the dose of midazolam (group A) and fentanyl (groups A and B) was observed (Additional file 1: Table S6).

**Table 3:** Number of prone sessions required according to the duration of the first prone session

	Total (*n* = 417)	Group A (*n* = 191)	Group B (*n* = 128)	Group C (*n* = 98)	*p* value
Number of sessions					0.398
1	318 (76.3)	137 (71.7)	100 (78.1)	81 (82.7)	
2	88 (21.1)	49 (25.7)	24 (18.8)	15 (15.3)	
3	9 (2.2)	4 (2.1)	3 (2.3)	2 (2)	
4	2 (0.5)	1 (0.5)	1 (0.8)	–	

Data are *n* (%). Group A patients remained 2–3 days in prone position during their first session. Group B patients remained 4–5 days in prone position during their first session. Group C patients remained more than 5 days in prone position during their first session

Regarding physiologic changes observed after starting prone position, PaO_2_:FiO_2_ ratio, static compliance and pH increased while driving pressure decreased (Table 2). By the end of the first prone session PaO_2_:FiO_2_ ratio was 231 (189–283) mm Hg.

The relative distribution of patients according to the first prone session duration is shown in Additional file 1: Fig. S3; 191 patients (46%) received a 2- to 3-day session (group A), 128 patients (31%) a 4- to 5-day session (group B), and 98 patients (24%) a session longer than 5 days (group C). There was no association between the first prone session duration and requirement of repeated prone sessions (Additional file 1: Table S7).

Before intubation, group C had a higher frequency of sepsis, higher use of HFNC and awake prone positioning, and lower PaO_2_:FiO_2_ ratio. After intubation, group C maintained lower PaO_2_:FiO_2_ ratio, higher PaCO_2_, ventilatory ratio, plateau and driving pressures, indicating greater severity of respiratory failure already before prone positioning (Additional file 1: Table S5).

**Fig. 1:**
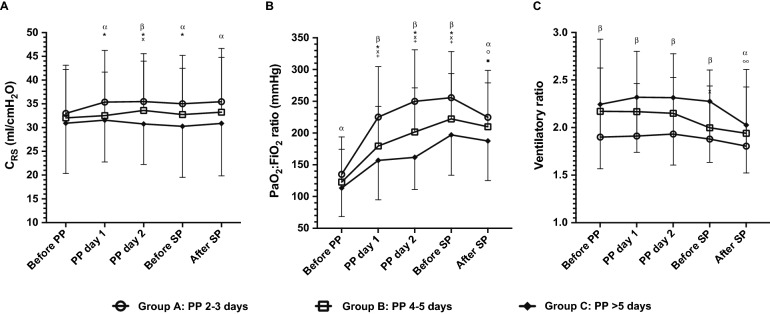
Respiratory system compliance, PaO2:FiO2 ratio and ventilatory ratio along prone positioning. Lines and symbols show mean and standard deviation of each variable before prone positioning (PP), at day 1 and at day 2 in prone, before supine position (SP) and after back to SP; C_RS_: respiratory system compliance. *, x, + : *p*-value < 0.001 for comparisons of different time points with their respective pre-PP values in patients from group A, B, and C, respectively.°, ▪, ∞: *p*-value < 0.001 for comparisons between before SP and after SP in patients from group **A** **B**, and **C**, respectively. α: *p*-value  < 0.01 for inter-group comparisons at different time points. β: p-value < 0.001 for inter-group comparisons at different time points

During prone positioning, PaO_2_:FiO_2_ ratio increased progressively in all 3 groups; but the increment was greater in group A. Static compliance increased only in group A, while ventilatory ratio remained rather stable in the 3 groups (Fig. 1 and Table 2). Return to supine position was associated with a significant decrease in PaO_2_:FiO_2_ ratio in groups A and B (Fig. 1).

### Safety and outcomes

Incidence of pressure sores is shown in Table 3. Grade 1–2 pressure sores in the ventral body surface were observed in 23.9% of patients during the first prone session, being higher the incidence in groups with longer prone sessions. No patient presented grade 3–4 pressure sores. Cumulative incidence of pressure sores up to day 7 was 36.2% in the whole cohort. There was an increased odd in Group C compared with group A (O.R = 1.734 [CI 95%: 1.044–2.879], *p*-value = 0.034). However, after controlling by SOFA score in logistic regression, the odds of pressure sores were similar among the three groups (O.R Group A = reference; O.R Group B = 0.794, *p*-value = 0.389; O.R Group C = 1.175, *p*-value = 0.564). Unplanned removal of vascular catheters and endotracheal tube complications were infrequent, without differences between groups (Table 4).

**Table 4:** Adverse events and clinical outcomes of prolonged continuous prone positioning

	Total (*n* = 417)	Group A (*n* = 191)	Group B (*n* = 128)	Group C (*n* = 98)	*p*-value
Adverse events, *n* (%)					
Pressure sores^a^, *n* (%)	97 (23.9)	27 (14.2)	29 (23.6)	41 (44.1)	< 0.001
Pressure sores up to day 7^b,^ *n* (%)	147 (36.2)	63 (33.2)	41 (33.3)	43 (46.2)	0.077
Vascular catheters displacement^a^, *n* (%)	11 (2.7)	4 (2.1)	3 (2.4)	4 (4.3)	0.535
Non-scheduled extubation^a^, *n* (%)	17 (4.2)	8 (4.2)	4 (3.3)	5 (5.4)	0.465
Endotracheal obstruction^a^, *n* (%)	14 (3.4)	5 (2.6)	4 (3.3)	5 (5.4)	0.728
Clinical outcomes					
90-day mortality, *n* (%)	151 (36.2)	58 (30.4)	46 (35.9)	47 (48)	0.014
ICU-mortality, *n* (%)	131 (31.4)	53 (27.8)	37 (28.9)	41 (41.8)	0.043
In-hospital mortality, *n* (%)	139 (33.3)	54 (28.3)	39 (30.5)	46 (46.9)	0.005
Tracheostomy, *n* (%)	80 (19.6)	20 (10.7)	24 (19)	36 (37.5)	< 0.001
Time on MV (days), median (IQR)	15 (10–22)	12 (8- 19)	16 (11–23)	21 (13–32)	< 0.001
ICU LoS (days), median (IQR)	17 (13–25)	15 (11–22)	18 (13–27)	22 (15–32)	< 0.001
Hospital LoS (days), median (IQR)	24 (16–38)	21 (14–33)	27 (16–40)	29 (16–50)	0.001

^a^Incidence relate to the first prone session

^b^Cumulative incidence up to day 7 (considering  ≥ 1 prone session)

Group A patients remained 2–3 days in prone position during their first session. Group B patients remained 4–5 days in prone position during their first session. Group C patients remained more than 5 days in prone position during their first session

*ICU* intensive care unit; MV: mechanical ventilation; LoS: length of stay

ICU and 90-day mortality were 31.4% and 36.2%, respectively (Table 4). Risk/protective factors for 90-day mortality in the unadjusted analysis are shown in Additional file 1: Table S10. Patients in group C presented higher 90-day mortality than patients from groups A and B (Table 4). However, after controlling for confounding factors, risk of death was similar among groups: group A (reference); group B HR = 1.297, 95% CI [0.811–2.075], *p*-value = 0.277; and group C HR = 1.390, 95% CI [0.855–2.261], *p*-value = 0.184.

Time on mechanical ventilation was 15 (10–22) days and 19.6% of patients required tracheostomy. Patients in group C had a longer duration of mechanical ventilation, ICU and hospital length of stay, and a higher rate of tracheostomy (Table 4).

## Discussion

In a large multicenter cohort of mechanically ventilated patients with moderate-to-severe COVID-19-related ARDS from Chile, we observed that a strategy of continuous prolonged prone positioning was feasible and safe. The main adverse event was low-grade pressure sores in the ventral body surface, but the rate of severe adverse events was low. Most patients required a single prone session that lasted 3 to 5 days, although in a subgroup it was extended beyond 5 days. Importantly, prone session duration was not independently associated to the risk of pressure sores.

A few single-center series of prolonged prone positioning had been previously reported; six before the COVID-19 pandemic (including 255 patients in total) and seven during the pandemic (including 162 patients in total). Only four series had more than 20 patients and the largest study included 116 ARDS patients from Korea. The median duration of prone sessions in these series lasted between 34 h and 3 days (Additional file 1: Table S12). Here, we report the largest cohort of ARDS patients treated with prolonged prone positioning reported up to now, including several heterogeneous centers, alongside highly granular data. Our study indicates that prolonged prone positioning can be implemented on a large scale, and that prone sessions can be extended for several days if required, without major side effects.

Prone session duration was highly variable according to the time each patient required to achieve the predefined PaO_2_:FiO_2_ ratio of 200, which contrasts with the standard approach of fix time-limited sessions. The underlying rationale was to turn patients back to supine position only after there was a strong assumption that prone position may no longer be required. According to the PROSEVA trial, repeated prone sessions are indicated if PaO_2_:FiO_2_ ratio falls below 150 once turned back to supine position [1]. As oxygen exchange worsens in most patients after returning to supine position [1, 8, 14], we defined a pragmatic criterion to predict whether the patient would be able to sustain a PaO_2_:FiO_2_ ratio above 150 in supine position: patients should reach a PaO_2_:FiO_2_ ratio above 200 while on prone position. Such criterion showed to be a reasonable predictor as no patient of the entire cohort who had reached this threshold exhibited a PaO_2_:FiO_2_ ratio below 150 after being turned back to supine position (Additional file 1: Fig. S4). Importantly, similar to the PROSEVA trial, PaO_2_:FiO_2_ ratio is used as a proxy of severity to determine when prone position may be justified, but not to identify “responders”. It has been shown that oxygenation response to prone position is not associated with its favorable impact on survival, which is presumed to be explained by enhanced lung protection [17, 30]. A potential gain of our approach compared to the conventional daily prone sessions is a lower chance of interrupting the protective effect of prone position, during a period in which it is still beneficial in patients who maintain a higher risk of ventilator-induced lung injury.

When analyzing the different cohorts of COVID-19 patients treated with prone positioning, there is a clear inverse relation between duration of prone sessions and number of sessions required (Additional file 1: Table S11). In our study, 76.3% of patients required a single prone session, which is consistent with a recent report in 61 COVID-19 patients treated with prolonged prone ventilation of whom 46 required a single session [26]. This contrasts with other series using daily prone sessions, which reported 3 to 4 sessions/patient on average (Additional file 1: Table S11). Several studies have reported that increased workload associated with repeated repositioning is one of the main factors which precludes implementation of prone positioning [1, 2, 31].

As previous reports of prolonged prone positioning are single-center studies, most including a small number of patients, there has been concern regarding the feasibility of implementing such strategy at a wider scale. In the present study most ICUs were heavily overloaded during the first COVID-19 pandemic wave and had to lower some nursing standards (Additional file 1: Table S2). Despite these adverse circumstances, and the heterogeneity in terms of organization and staffing, all participating ICUs were able to successfully implement continuous prolonged prone positioning as their standard protocol. Almost two-thirds of mechanically ventilated patients in the participating centers received prolonged prone positioning and, in most patients, it was started on the first day of ventilation, which has been associated to better outcomes [8, 10]. It is likely that a protocol perceived as less demanding contributed to a broad and timely implementation of prone positioning.

Prone position-related adverse effects were uncommon except for pressure sores on the ventral body surface, with a rate comparable to that reported in the PROSEVA trial [1, 27]. No patient developed grade 3–4 pressure sores or required surgical debridement; this is consistent with several previous studies of prone positioning which have shown that most prone-related pressure sores are grade 1–2 [15, 26, 28, 29]. Previous experiences with prolonged prone position published up to now had reported a rate of pressure sores ranging between 13.3 and 67%, with lower than 5% of the cases corresponding to grade 3 (Additional file 1: Table S12). Non-scheduled extubation, endotracheal tube obstruction and displacement of vascular catheters were infrequent, with lower rates compared to those reported in previous studies using intermittent daily prone positioning [1, 15]. This finding may be related to the intrinsic risk associated to the maneuver of changing position to and from prone positioning. Other major complications leading to interruption of prone sessions were also observed at lower rates than previously reported [1].

As a relevant proportion of patients required very long prone sessions beyond what we had seen in the past [18, 20], we decided to separate the cohort in groups to better analyze the potential impact of this variable on safety. We observed that group C had a higher rate of pressures sores and a larger mortality than groups A and B. However, after adjusting for confounding factors, multivariable analyses revealed that duration of prone sessions was not independently associated with pressure sores or mortality. Because patients from group C presented higher use of HFNC and awake prone, and higher severity of lung disease once intubated, we cannot rule out whether a delayed intubation affected the progressive course of the disease and contributed to lung loss of aeration and/or fibrotic organization in these patients; there was no provision of a standardized approach to intubation decisions.

Our study certainly has limitations. First, it was retrospective which has well known limitations compared to a prospective design. However, prospectively collecting the amount of granular data presented in this study would have been extremely challenging in the context of overwhelmed ICUs. Second, due to time and resource constraints, we studied a convenience sample using an 8-week enrollment window, which may be affected by selection bias. However, no major differences were observed between the individual mortality rates of the participating ICUs during the first wave in Chile, with the corresponding mortality in the study sample (Additional file 1: Table S1). Third, as our study was limited to Chile in which most centers had previous experience with prolonged prone positioning, our findings cannot be generalized to places with less experience in this technique. Finally, we did not include a control group of patients treated with the conventional daily prone sessions, so we cannot compare both strategies.

## Conclusions

In conclusion, a nationwide strategy of continuous prolonged prone positioning was feasible with 3 out of 4 patients requiring a single uninterrupted session. Minor pressure sores in the ventral surface were frequent but within the ranges previously described for prone positioning, while severe adverse events were infrequent. By decreasing workload, this strategy may facilitate widespread use of prone positioning, one of the few life-saving interventions for ARDS.

## Supplementary Information

**Additional file 1. Table S1.** Data from Participating Centers during the First wave of the COVID-19 pandemic in Chile (between April 1st and August 31st). **Table S2.** Organizational variables and prone positioning management before and at the first Covid-19 outbreak in the participating centers. **Table S3.** Counts of missing data. **Table S4.** Observed characteristics between participants with complete and incomplete data. **Table S5.** Respiratory and hemodynamic characteristics of patients before and after intubation. **Table S6.** Sedative agents, Opioids and Neuromuscular Blockade before and after prone position initiation. **Table S7.** Description of prone positioning sessions. **Table S8.** Reasons for interrupting prone sessions. **Table S9.** Variables associated to groups classification according to the duration of the first prone session. **Table S10.** Risks factors for 90-day mortality. **Table S11.** Large cohort studies of Covid-19 patients treated with mechanical ventilation and prone positioning. **Table S12.** Case series of prolonged prone positioning. **Figure S1.** Algorithm for the management of COVID-19 patients with Acute Respiratory Failure (Chilean Society of Intensive Care Medicine). **Figure S2.** Cohort flowchart for patients treated with prolonged prone positioning. **Figure S3.** Distribution of patients according to the first prone session duration (days). **Figure S4.** Correlation between PaO_2_:FiO_2_ ratio in prone before supine and PaO_2_:FiO_2_ ratio in supine after prone. **Figure S5.** Directed acyclic graph to select the confounding factors.

## Data Availability

The data that support the findings of this study are available from the corresponding authors, RC and AB, upon reasonable request.

## Abbreviations

ARDS: Acute respiratory distress syndrome
COVID-19: Coronavirus disease 2019
FiO_2_: Fraction of inspired oxygen
HFNC: High-flow nasal cannula
IBW: Ideal body weight
ICU: Intensive Care Unit
IQR: Interquartile range
NPUAP: National Pressure Ulcer Advisory Panel's updated pressure ulcer staging system
PaCO_2_: Arterial partial pressure of carbon dioxide
PaO_2_: Arterial partial pressure of oxygen
PEEP: Positive end-expiratory pressure
