# Supplementary material for: Continuous prolonged prone positioning in COVID-19-related ARDS: a multicenter cohort study from Chile
Source: Ann Intensive Care. 2022 Nov 28;12:109. doi: 10.1186/s13613-022-01082-w (PMC9702866; doi:10.1186/s13613-022-01082-w)
Supplement: Supplementary file 1 — Additional file 1. Table S1. Data from Participating Centers during the First wave of the COVID-19 pandemic in Chile (between April 1st and August 31st). Table S2. Organizational variables and prone positioning management before and at the first Covid-19 outbreak in the participating centers. Table S3. Counts of missing data. Table S4. Observed characteristics between participants with complete and incomplete data. Table S5. Respiratory and hemodynamic characteristics of patients before and after intubation. Table S6. Sedative agents, Opioids and Neuromuscular Blockade before and after prone position initiation. Table S7. Description of prone positioning sessions. Table S8. Reasons for interrupting prone sessions. Table S9. Variables associated to groups classification according to the duration of the first prone session. Table S10. Risks factors for 90-day mortality. Table S11. Large cohort studies of Covid-19 patients treated with mechanical ventilation and prone positioning. Table S12. Case series of prolonged prone positioning. Figure S1. Algorithm for the management of COVID-19 patients with Acute Respiratory Failure (Chilean Society of Intensive Care Medicine). Figure S2. Cohort flowchart for patients treated with prolonged prone positioning. Figure S3. Distribution of patients according to the first prone session duration (days). Figure S4. Correlation between PaO2:FiO2 ratio in prone before supine and PaO2:FiO2 ratio in supine after prone. Figure S5. Directed acyclic graph to select the confounding factors. [file 13613_2022_1082_MOESM1_ESM.docx]

**ADDITIONAL INFORMATION**

**ssss**

**Continuous prolonged prone positioning in COVID-19-related ARDS: A multicenter cohort study from Chile**

Rodrigo A. Cornejo, Jorge Montoya, Abraham IJ Gajardo, Jerónimo Graf, Leyla Alegría, Romyna Baghetti, Anita Irarrázaval, César Santis, Nicolás Pavez, Sofía Leighton, Vinko Tomicic, Daniel Morales, Carolina Ruiz, Pablo Navarrete, Patricio Vargas, Roberto Gálvez, Victoria Espinosa, Marioli Lazo, Rodrigo A. Pérez-Araos, Osvaldo Garay, Patrick Sepúlveda, Edgardo Martinez, Alejandro Bruhn on behalf of the SOCHIMI Prone-COVID-19 Group.

**Corresponding authors:**

Rodrigo A. Cornejo MD, FACP. Full Professor Universidad de Chile. Unidad de Pacientes Críticos, Departamento de Medicina, Hospital Clínico Universidad de Chile. Dr. Carlos Lorca Tobar 999, 2º piso, Independencia, Santiago, Chile. E-mail: [racornej@gmail.com](mailto:racornej@gmail.com). Mobile phone: +56988399280.

Postal code: 8380456

Alejandro Bruhn, M.D, Ph.D. Full Professor Departamento de Medicina Intensiva, Edificio Académico Escuela de Medicina, Pontificia Universidad Católica de Chile, Diagonal Paraguay 362, 6º piso, Santiago, Chile. P.O. Box 114D. Email: [alejandrobruhn@gmail.com](mailto:alejandrobruhn@gmail.com). Mobile phone +56983260734. Postal code: 8330077

**Table of Contents:**

Collaborators………………………………………………………………………………3

**Tables**

*Table S1.* Data from Participating Centers during the First wave of the COVID-19 pandemic in Chile (between April 1st and August 31st)…………………………………..5

*Table S2.* Organizational variables and prone positioning management before and at the first Covid-19 outbreak in the participating centers……………………………………….6

*Table S3.* Counts of missing data………………………………………………………….9

*Table S4.* Observed characteristics between participants with complete and incomplete data.……...………...……………………………………………………………………...12

*Table S5.* Respiratory and hemodynamic characteristics of patients before and after intubation………………………………….........................................................................16

*Table S6.* Sedative agents, Opioids and Neuromuscular Blockade before and after prone position initiation………………………………………………………………………….18

*Table S7*. Description of prone positioning sessions……………………………………...19

*Table S8*. Reasons for interrupting prone sessions………………………………………..20

*Table S9.* Variables associated to groups classification according to the duration of the first prone session………………………………………………………………………………21

*Table S10.* Risks factors for 90-days mortality..…………………………………………..22

*Table S11*. Large cohort studies of Covid-19 patients treated with mechanical ventilation and prone positioning...………………………………………………………...................24

*Table S12*. Case series of prolonged prone positioning…………………………………..25

**Figures**

# *Figure S1.* Algorithm for the management of COVID-19 patients with Acute Respiratory Failure (Chilean Society of Intensive Care Medicine)……………………………………26

# *Figure S2*. Cohort flowchart for patients treated with prolonged prone positioning……..27

*Figure S3.* Distribution of patients according to the first prone session duration (days)…28

*Figure S4.* Correlation between PaO_2_:FiO_2_ ratio in prone before supine and PaO_2_:FiO_2_ ratio in supine after prone………………………………………………………………………29

*Figure S5.* Directed acyclic graph to select the confounding factors……………………..30

**References**………………………………………………………………………………..31**Collaborators:**

Assisted in patient care and data collection for patients with coronavirus disease 2019 (COVID-19) treated with prolonged prone positioning following the national recommendations from Chilean Society of Intensive Care Medicine (SOCHIMI) and entered data into the SOCHIMI Prone-COVID-19 Group registry.

Nicole Rossel^1^, María José Martin^1^, Juan Nicolás Medel^1^, Vanessa Oviedo^2^, Magdalena Vera^2^, Vicente Torres^3^, José Miguel Montes^4^, Álvaro Salazar^4^, Carla Muñoz^5^, Francisca Tala^6^, Mariana Migueles^6^, Claudia Ortiz^7^, Felipe Gómez^8^, Luis Contreras^8^, Itzia Daviu^9^, Yurimar Rodriguez^9^, Carol Ortiz^10^, Andrés Aquevedo^2,11^, Rodrigo Parada^12^, Cristián Vargas^13^, Miguel Gatica^14^, Dalia Guerrero^14^, Araceli Valenzuela^14^, Diego Torrejón^15^.

**Affiliations:** ^1^Unidad de Pacientes Críticos, Departamento de Medicina, Hospital Clínico Universidad de Chile, Santiago, Chile; ^2^Departamento de Medicina Intensiva, Facultad de Medicina, Pontificia Universidad Católica de Chile, Santiago, Chile; ^3^Unidad de Pacientes Críticos Adultos, Hospital Van Buren, Valpariso, Chile; ^4^Departamento de Paciente Crítico, Clínica Alemana de Santiago, Chile; ^5^Unidad de Paciente Crítico, Hospital La Serena, Coquimbo, Chile; ^6^Unidad de Pacientes Críticos, Hospital Barros Luco Trudeau, Santiago, Chile; ^7^Unidad de Pacientes Críticos, Hospital Regional de Concepción, Concepción, Chile; ^8^Unidad de Pacientes Críticos, Hospital Padre Hurtado, Santiago, Chile; ^9^Unidad de Paciente Crítico, Hospital Clinico Regional de Antofagasta, Antofagasta, Chile; ^10^Unidad de Pacientes Críticos, Hospital Clínico Dra. Eloisa Diaz I - La Florida, Santiago, Chile; ^11^Unidad de Paciente Crítico, Complejo Asistencial Dr. Sótero del Río, Santiago, Chile; ^12^Unidad de Pacientes Críticos, Hospital Clínico Herminda Martín, Chillán, Chile; ^13^Hospital del Salvador, Departamento de Medicina Interna, Universidad de Chile, Santiago, Chile; ^14^Unidad de Pacientes Críticos, Hospital Regional de Iquique, Iquique, Chile; ^15^Unidad de Paciente Crítico, Clínica Alemana de Temuco, Temuco, Chile.

**Table S1. Data from participating centers during the first wave of the COVID-19 pandemic in Chile (between April 1^st^ and August 31^th^)**

| **Centers’ characteristics** | **Frequency (n) or median (IQR)** | |
| --- | --- | --- |
| *Total population in participating centers* | | |
| Type of center | | 11 Public  2 Private  2 University |
| Total number of ventilated patients, n | | 2822 |
| Ventilated patients by ICU, median (IQR) | | 156 (98-334) |
| Total deaths in ventilated patients, n | | 969 |
| Hospital mortality in ventilated patients by ICU (%), median (IQR) | | 31 (19-42) |
| Total number of patients ventilated in prone position, n | | 1795 |
| Patients ventilated in prone position by ICU, median (IQR) | | 105 (56-209) |
| Total hospital deaths in patients ventilated in prone position, n | | 687 |
| Hospital mortality in patients ventilated in prone position by ICU (%), median (IQR) | | 32 (29-45) |
| ***Study population*** | | |
| Total number of patients ventilated in prone position, n | | 417 |
| Patients ventilated in prone position by ICU, median (IQR) | | 21 (15-43) |
| Total hospital deaths in patients ventilated in prone position, n | | 139 |
| Hospital mortality in ventilated in prone position by ICU (%), median (IQR) | | 38 (20-44) |

Between March 3 (the first case of COVID-19 in Chile) and August 31^st^, 2020, there were 444921 COVID-19 positive cases older than 15 years; 7792 of them were mechanically ventilated patients with COVID-19 in our country.(1)

**Table S2. Organizational variables and prone positioning management before and at the first COVID-19 outbreak in the participating centers**

|  | **Before COVID-19 pandemic** | **Peak of first COVID-19 outbreak** |
| --- | --- | --- |
| ***I. Organizational variables*** |  |  |
| **About the hospitals** |  |  |
| Total number of Hospital beds | 7814 | 7254 |
| Total number of Intensive care beds | 292* | 920 |
| Total number of Intermediate care beds | 445 | 573 |
| **About the participating ICUs** |  |  |
| Number of intensive care beds | 250 | 635 |
| Patient to registered nurse ratio**- number of ICUs (%) |  |  |
| 2:1 | 2 (13) | 2 (13) |
| 3:1 | 11 (73) | 11 (73) |
| 4:1 | 2 (13) | 2 (13) |
| Patient to physician ratio - number of ICUs (%) |  |  |
| 6:1 | 7 (47) | 6 (40) |
| 7:1 | 4 (27) | 4 (27) |
| 8:1 | 3 (20) | 4 (27) |
| 9:1 | 1 (7) | 1 (7) |
| DT intensivist physician staffing - number of ICUs (%) | 14 (93) | 13 (87) |
| Type of ICU mattress in most beds - number of ICUs (%) |  |  |
| Standard (spring) | - | - |
| Viscoelastic foam | 7 (47) | 8 (53) |
| Alternating air pressure | 6 (40) | 6 (40) |
| Air fluidized | 2 (13) | 1 (7) |
| ICU Physiotherapist - number of ICUs (%) |  |  |
| Not available | 1 (7) | 1 (7) |
| Only daytime | 3 (20) | 2 (13) |
| Continuous | 11 (73) | 12 (80) |
| Portable X-ray available - number of ICUs (%) | 15 (100) | 15 (100) |
| CT scan available - number of ICUs (%) | 15 (100) | 15 (100) |
| Fiberoptic bronchoscopy available - number of ICUs (%) | 13 (87) | 14 (93) |
| Sedation protocol - number of ICUs (%) |  |  |
| No written protocol | 1 (7) | 1 (7) |
| Daily suspension | 2 (13) | 2 (13) |
| Titration according to scales | 13 (87) | 13 (87) |
| ***II. Approach to prone positioning*** |  |  |
| How often did you use prolonged prone positioning (>48 h) before Covid-19 pandemics?  Likert scale 1-5; mean ± SD | 3.6 ± 1.3 | - |
| ICU has a written protocol / checklist for prone positioning - number of ICUs (%) | 11 (73) | 13 (87) |
| Nursing staff has received specific training for prone positioning  Likert scale 1-5; mean ± SD | 3.7±1.2 | 4.6±0.9 |
| Is there a formal leader who directs patient repositioning? - number of ICUs (%) | 15 (100) | 15 (100) |
| What position does the ICU use for placing patients in prone position? - number of ICUs (%) |  |  |
| Swimming | 15 (100) | 15 (100) |
| Other | - | - |
| How many staff participate in patient repositioning? - number of persons - mean ± SD | 5±1 | 5±1 |
| How many staff participate in patient lateralization? - number of persons - mean ± SD | 3±1 | 3±1 |
| Frequency of lateralization used in the ICU - number of ICUs (%) |  |  |
| Hourly | 1 (7) | 1 (7) |
| Every 2 hours | 7 (47) | 6 (40) |
| Every 3 or 4 hours | 7 (47) | 8 (53) |
| Use of dressings to offload pressure points - number of ICUs (%) |  |  |
| None | - | - |
| Transparent film | 4 (27) | 4 (27) |
| Hydrocolloids | 8 (53) | 8 (53) |
| Other | 3 (20) | 3 (20) |
| Do you use dressings or ointments to protect eyes? - number of ICUs (%) | 11 (73) | 11 (73) |
| How is the tracheal tube fixed? - number of ICUs (%) |  |  |
| Adhesive tape | 11 (73) | 12 (80) |
| Fixator | 4 (27) | 3 (20) |
| Bed inclination while on prone position - number of ICUs (%) |  |  |
| Flat | - | - |
| 5 to 15º | 15 (100) | 15 (100) |
| Use of special positioning devices to support prone positioning (can answer > 1) - number of ICUs (%) |  |  |
| Standard pillows | 12 (80) | 10 (67) |
| Special cushions for prone positioning | 2 (13) | 5 (33) |
| Donut pillow for eye & nose protection | 8 (53) | 9 (60) |
| Rotational bed | - | - |
| Other | - | - |
| Was tracheal tube repositioned side-to-side daily? - number of ICUs (%) | 14 (93) | 13 (87) |
| Routine assessment of tracheal tube cuff pressure - number of ICUs (%) | 15 (100) | 15 (100) |
| Do you use subglottic aspiration?  Likert scale 1-5; mean ± SD | 3±1.8 | 3.4±1.9 |
| A closed suction system was used to remove airway secretions  Likert scale 1-5; mean ± SD | 4.5±0.9 | 5±0 |
| How often do physiotherapists perform passive mobilization of extremities while on prone position  Likert scale 1-5; mean ± SD | 4.3 ± 1.3 | 4.1 ± 1.1 |
| Use of daily baths - number of ICUs (%) | 15 (100) | 15 (100) |
| Skin moisturizing regimen - number of ICUs (%) |  |  |
| 1/day | 2 (13) | 2 (13) |
| 2/day | 9 (60) | 10 (67) |
| 3/day | 2 (13) | 1 (7) |
| 4/day | 2 (13) | 2 (13) |
| Use of capnography while on prone position  Likert scale 1-5; mean ± SD | 2.7±1.6 | 2.7±1.4 |
|  |  |  |
| ICU: Intensive care unit, CT: computed tomography, DT: Daytime.  * Some hospitals had more than one ICU and some of these ICUs did not participate in the study | | |
| ** In Chile nursing assistants support the work of ICU registered nurses in a ratio of one per ≈ 3 ICU patients. | | |

**Table S3. Counts of missing data**

| **Variable** | | | **Missing, n (%)** |
| --- | --- | --- | --- |
| ***Basal characteristics*** | | | |
| Age | | | 1 |
| Sex | | | 0 |
| Body mass index | | | 0 |
| Hypertension | | | 0 |
| Type 2 diabetes mellitus | | | 0 |
| Coronary heart disease | | | 0 |
| Chronic liver disease | | | 0 |
| Immunosuppression | | | 0 |
| Chronic kidney disease | | | 0 |
| Obesity | | | 0 |
| Other | | | 0 |
| None | | | 0 |
| APACHE II score | | | 47 |
| SOFA score | | | 62 |
| Sepsis | | | 5 |
| Days from-to | | |  |
| Diagnosis to hospital admission | | 33 |  |
| Hospital to ICU admission | | 0 |  |
| Diagnosis to invasive ventilation | | 33 |  |
| Invasive ventilation to prone position | | 0 |  |
|  | | | |
| ***Before intubation*** | | | |
| Awake patient positioning | | | 30 |
| Ventilatory support | | | 45 |
| Respiratory rate | | | 57 |
| PaO_2_ | | 64 | |
| FiO_2_ | | 80 | |
| PaO_2_:FiO_2_ ratio | | 80 | |
| PaCO_2_ | | 66 | |
| pH | | 67 | |
| Mean arterial pressure | | 54 | |
| Heart rate | | 50 | |
| Vasoactive support | | 118 | |
|  | | | |
| ***After intubation*** | | | |
| Patient positioning | | 24 | |
| Mechanical ventilation mode | | 20 | |
| Neuromuscular blockage | | 25 | |
| Respiratory rate | | 28 | |
| Tidal volume | | 27 | |
| Plateau pressure | | 73 | |
| Driving pressure | | 72 | |
| PEEP | | 26 | |
| Static compliance | | 75 | |
| PaO_2_ | | 31 | |
| FiO_2_ | | 21 | |
| PaO_2_:FiO_2_ ratio | | 31 | |
| PaCO_2_ | | 33 | |
| pH | | 34 | |
| Ventilatory ratio | | 69 | |
| Mean arterial pressure | | 43 | |
| Heart rate | | 39 | |
| Vasoactive support | | 60 | |
|  | | | |
| ***Before starting prone position*** | | | |
| Mechanical ventilation mode | | 13 | |
| Neuromuscular blockage | | 13 | |
| Respiratory rate | | 7 | |
| Tidal volume | | 33 | |
| Plateau pressure | | 53 | |
| Driving pressure | | 53 | |
| PEEP | | 8 | |
| Static compliance | | 54 | |
| PaO_2_ | | 9 | |
| FiO_2_ | | 4 | |
| PaO_2_:FiO_2_ ratio | | 9 | |
| PaCO_2_ | | 10 | |
| pH | | 10 | |
| Ventilatory ratio | | 43 | |
| Mean arterial pressure | | 22 | |
| Heart rate | | 21 | |
| Vasoactive support | | 19 | |
|  | |  | |
| ***At day 1 in prone positioning*** | | | |
| Mechanical ventilation mode | | | 2 |
| Neuromuscular blockage | | | 3 |
| Respiratory rate | | | 0 |
| Tidal volume | | | 26 |
| Plateau pressure | | | 23 |
| Driving pressure | | | 23 |
| PEEP | | | 0 |
| Static compliance | | | 23 |
| PaO_2_ | | | 1 |
| FiO_2_ | | | 0 |
| PaO_2_:FiO_2_ ratio | | | 1 |
| PaCO_2_ | | | 1 |
| pH | | | 1 |
| Ventilatory ratio | | | 27 |
| Mean arterial pressure | | | 1 |
| Heart rate | | | 1 |
| Vasoactive support | | | 8 |
|  | | |  |
| ***At day 2 in prone positioning*** | | | |
| Mechanical ventilation mode | | | 1 |
| Neuromuscular blockage | | | 5 |
| Respiratory rate | | | 2 |
| Tidal volume | | | 30 |
| Plateau pressure | | | 22 |
| Driving pressure | | | 22 |
| PEEP | | | 2 |
| Static compliance | | | 22 |
| PaO_2_ | | | 0 |
| FiO_2_ | | | 1 |
| PaO_2_:FiO_2_ ratio | | | 1 |
| PaCO_2_ | | | 0 |
| pH | | | 0 |
| Ventilatory ratio | | | 30 |
| Mean arterial pressure | | | 2 |
| Heart rate | | | 2 |
| Vasoactive support | | | 28 |
|  | | |  |
| ***Physiologic variables before and after supine position*** | | |  |
| PaO_2_:FiO_2_ ratio before supine position | | 20 | |
| PaO2:FiO2 ratio after supine position | | 29 | |
| Ventilatory ratio before supine position | | 48 | |
| Ventilatory ratio after supine position | | 61 | |
| Static compliance before supine position | | 65 | |
| Static compliance after supine position | | 80 | |
|  | |  | |
| ***Outcomes*** | | | |
| Deaths at ICU | | 0 | |
| Deaths at hospital | | 0 | |
| Deaths at 90-days | | 0 | |
| Pressure sores | | 11 | |
| Vascular catheters displacement | | 11 | |
| Non-schedule extubation | | 11 | |
| Endotracheal obstruction | | 11 | |
| ICU length of stay | | 6 | |
| Hospital length of stay | | 18 | |
| SOFA, sequential organ failure assessment; APACHE, acute physiology and chronic health evaluation PaO_2_: partial pressure of arterial oxygen, FiO_2_: Inspired oxygen fraction, PaO_2_:FiO_2_ ratio: ratio of partial pressure of arterial oxygen to inspired oxygen fraction, PaCO_2_: partial pressure of arterial carbon dioxide, PEEP: Positive end-expiratory pressure, Static compliance: Static respiratory system compliance, Ventilatory ratio is a unit less index calculated as (minute ventilation in mL/min x PaCO_2_) / (Ideal body weight x 100 x 37.5). | | | |

**Table S4. Observed variables in patients with incomplete and complete data for the main analysis**

|  | **Incomplete data  (n = 107)** | **Complete data  (n = 310)** | **p-value** |
| --- | --- | --- | --- |
| ***Basal characteristics*** | | | |
| Age, median (IQR) | 59.0 (48.0, 69.0) | 62.0 (53.0, 68.0) | 0.22 |
| Male sex | 85 (79.4%) | 216 (69.7%) | 0.060 |
| Body mass index, median (IQR) | 29.4 (26.5, 32.3) | 30.1 (26.7, 34.6) | 0.31 |
| Comorbidities, n(%) |  |  |  |
| Hypertension | 52 (48.6%) | 180 (58.1%) | 0.092 |
| Type 2 diabetes mellitus | 39 (36.4%) | 127 (41.0%) | 0.43 |
| Coronary heart disease | 3 (2.8%) | 14 (4.5%) | 0.58 |
| Chronic liver disease | 3 (2.8%) | 7 (2.3%) | 0.72 |
| Immunosuppression | 0 (0.0%) | 6 (1.9%) | 0.35 |
| Chronic kidney disease | 5 (4.7%) | 15 (4.8%) | 1.00 |
| Obesity | 27 (25.2%) | 120 (38.7%) | 0.014 |
| Other | 31 (29.0%) | 90 (29.0%) | 1.00 |
| None | 20 (18.7%) | 43 (13.9%) | 0.27 |
| APACHE II score, median (IQR) | 13.0 (10.0, 16.0) | 14.0 (10.0, 18.0) | 0.15 |
| SOFA score, median (IQR) | 4.0 (3.0, 6.0) | 5.0 (4.0, 7.0) | 0.021 |
| Sepsis | 13 (12.3%) | 87 (28.4%) | <0.001 |
| Days from-to, median (IQR) |  |  |  |
| Diagnosis to hospital admission | 0.0 (0.0, 3.0) | 0.0 (0.0, 3.0) | 0.77 |
| Hospital to ICU admission | 1.0 (0.0, 4.0) | 1.0 (0.0, 3.0) | 0.57 |
| Diagnosis to invasive ventilation | 3.0 (1.0, 7.0) | 3.0 (1.0, 6.0) | 0.77 |
| Invasive ventilation to prone position | 1.0 (0.0, 2.0) | 1.0 (0.0, 1.0) | 0.14 |
| Prone position session, days  median (IQR) | 3.0 (3.0, 5.0) | 4.0 (3.0, 6.0) | 0.009 |
| Groups of prone positioning |  |  |  |
| 2-3 days | 60 (56.1%) | 131 (42.3%) | 0.028 |
| 4-5 days | 30 (28.0%) | 98 (31.6%) |  |
| > 5 days | 17 (15.9%) | 81 (26.1%) |  |
| ***Before intubation*** | | | |
| Awake prone positioning | 14 (15.2%) | 98 (33.2%) | <0.001 |
| Ventilatory support, n(%) |  |  | 0.002 |
| Usual nasal cannula | 6 (6.7%) | 7 (2.5%) |  |
| Venturi mask | 41 (45.6%) | 139 (49.3%) |  |
| Non-Rebreather mask | 9 (10.0%) | 12 (4.3%) |  |
| High flow nasal cannula | 20 (22.2%) | 102 (36.2%) |  |
| Non-invasive ventilation | 13 (14.4%) | 22 (7.8%) |  |
| Respiratory rate, median (IQR) | 28.5 (24.0, 35.0) | 30.0 (27.0, 35.0) | 0.14 |
| Mean arterial pressure, median (IQR) | 87.0 (80.0, 97.0) | 88.0 (80.0, 96.0) | 0.91 |
| Heart rate, median (IQR) | 92.0 (75.0, 104.0) | 93.0 (80.0, 104.0) | 0.35 |
| Vasoactive support, n (%) | 7 (11.7%) | 18 (7.5%) | 0.30 |
| FiO_2_ %, median (IQR) | 80.0 (50.0, 100.0) | 80.0 (60.0, 95.0) | 0.19 |
| PaO_2_ mm Hg, median (IQR) | 65.8 (58.5, 75.3) | 65.7 (57.0, 80.0) | 0.64 |
| PaCO_2_, mm Hg, median (IQR) | 35.0 (32.0, 40.4) | 35.0 (31.2, 39.5) | 0.94 |
| pH, median (IQR) | 7.4 (7.4, 7.5) | 7.4 (7.4, 7.5) | 0.95 |
| paO2:FiO2 ratio | 89.3 (66.5, 140.5) | 85.8 (67.7, 118.2) | 0.33 |
| ***After intubation*** | | | |
| Supine positioning | 82 (84.5%) | 278 (93.9%) | 0.006 |
| Volume assist/control mode | 97 (98.0%) | 293 (98.3%) | >0.999 |
| Respiratory rate, median (IQR) | 25.0 (22.0, 28.0) | 25.0 (22.0, 28.0) | 0.89 |
| Plateau pressure, median (IQR) | 23.0 (21.0, 25.0) | 24.0 (21.0, 26.0) | 0.87 |
| Mean arterial pressure, median (IQR) | 80.0 (70.5, 88.5) | 77.0 (70.0, 88.0) | 0.64 |
| Heart rate, median (IQR) | 85.0 (75.0, 100.0) | 85.0 (73.0, 100.0) | 0.89 |
| Vasoactive support, median (IQR) | 54 (70.1%) | 202 (72.1%) | 0.78 |
| PaO_2_:FiO_2_ ratio, median (IQR) | 124.8 (92.0, 174.1) | 119.6 (82.9, 162.5) | 0.25 |
| Ventilatory ratio, median (IQR) | 1.8 (1.5, 2.3) | 1.9 (1.5, 2.4) | 0.29 |
| PaO_2_ mm Hg, median (IQR) | 83.5 (69.7, 96.4) | 80.0 (68.8, 95.2) | 0.57 |
| PaCO_2_, mm Hg, median (IQR) | 44.4 (38.2, 51.3) | 46.7 (38.7, 55.0) | 0.17 |
| pH, median (IQR) | 7.3 (7.3, 7.4) | 7.3 (7.2, 7.4) | 0.82 |
| Respiratory rate, median (IQR) | 25.0 (22.0, 28.0) | 25.0 (22.0, 28.0) | 0.89 |
| PEEP, median (IQR) | 10.0 (10.0, 12.0) | 10.0 (8.0, 12.0) | 0.72 |
| Driving pressure, median (IQR) | 12.0 (10.0, 14.0) | 12.0 (11.0, 15.0) | 0.77 |
| Static compliance, median (IQR) | 32.5 (25.5, 37.0) | 31.0 (26.0, 37.0) | 0.49 |
| PaO_2_:FiO_2_ ratio, median (IQR) | 124.8 (92.0, 174.1) | 119.6 (82.9, 162.5) | 0.25 |
| ***Before prone positioning*** | | | |
| Neuromuscular blockage, n (%) | 83 (81.4%) | 245 (81.1%) | >0.999 |
| Respiratory rate, median (IQR) | 26.0 (23.0, 28.0) | 26.0 (23.0, 30.0) | 0.38 |
| Tidal volume, median (IQR) | 6.1 (5.5, 6.5) | 6.1 (5.7, 6.8) | 0.32 |
| Plateau pressure, median (IQR) | 23.0 (21.0, 25.0) | 23.0 (21.0, 27.0) | 0.69 |
| Driving pressure, median (IQR) | 12.5 (10.0, 14.0) | 12.0 (11.0, 15.0) | 0.63 |
| Static compliance, median (IQR) | 31.5 (25.0, 38.0) | 31.0 (25.0, 38.0) | 0.99 |
| PEEP, median (IQR) | 10.0 (10.0, 12.0) | 10.0 (8.0, 12.0) | 0.97 |
| PaO_2_ mm Hg, median (IQR) | 75.2 (64.8, 90.0) | 74.4 (64.0, 86.0) | 0.77 |
| PaCO_2_, mm Hg, median (IQR) | 43.9 (39.9, 52.0) | 46.0 (39.0, 54.0) | 0.35 |
| pH, median (IQR) | 7.3 (7.3, 7.4) | 7.3 (7.3, 7.4) | 0.089 |
| Mean arterial pressure, median (IQR) | 80.0 (71.0, 90.0) | 77.0 (70.0, 87.0) | 0.15 |
| Heart rate, median (IQR) | 82.0 (70.0, 96.0) | 82.0 (70.0, 99.0) | 0.75 |
| Vasoactive support, median (IQR) | 61 (61.6%) | 224 (74.9%) | 0.014 |
| PaO_2_:FiO_2_ ratio, median (IQR) | 125.5 (87.5, 168.9) | 116.7 (84.6, 149.2) | 0.11 |
| Ventilatory ratio, median (IQR) | 1.8 (1.5, 2.2) | 2.0 (1.6, 2.5) | 0.056 |
| ***Prone positioning day 1*** | | | |
| Neuromuscular blockage, n (%) | 101 (95.3%) | 300 (97.4%) | 0.33 |
| Respiratory rate, median (IQR) | 26.0 (23.0, 28.0) | 27.0 (24.0, 30.0) | <0.001 |
| Tidal volume, median (IQR) | 6.0 (5.5, 6.5) | 6.3 (5.7, 6.9) | 0.074 |
| Plateau pressure, median (IQR) | 22.0 (20.0, 25.0) | 22.0 (21.0, 25.0) | 0.72 |
| Driving pressure, median (IQR) | 12.0 (10.0, 14.0) | 12.0 (10.0, 14.0) | 0.87 |
| Static compliance, median (IQR) | 31.0 (27.0, 38.0) | 33.0 (27.0, 39.0) | 0.72 |
| PEEP, median (IQR) | 10.0 (8.0, 12.0) | 10.0 (8.0, 12.0) | 0.76 |
| PaO_2_ mm Hg, median (IQR) | 83.2 (72.9, 102.0) | 83.9 (72.0, 102.0) | 0.82 |
| PaCO_2_, mm Hg, median (IQR) | 46.0 (40.9, 52.0) | 45.0 (40.0, 51.0) | 0.35 |
| pH, median (IQR) | 7.4 (7.3, 7.4) | 7.3 (7.3, 7.4) | 0.21 |
| Mean arterial pressure, median (IQR) | 78.0 (70.0, 87.0) | 78.0 (71.0, 85.0) | 0.67 |
| Heart rate, median (IQR) | 80.5 (72.0, 92.0) | 80.0 (69.0, 92.0) | 0.74 |
| Vasoactive support, median (IQR) | 69 (69.7%) | 246 (79.4%) | 0.055 |
| PaO_2_:FiO_2_ ratio, median (IQR) | 190.0 (138.0, 255.8) | 183.9 (137.4, 237.6) | 0.39 |
| Ventilatory ratio, median (IQR) | 1.8 (1.6, 2.3) | 2.0 (1.7, 2.6) | 0.007 |
| ***Physiologic variables*** | | | |
| Static compliance at day 2 in prone positioning, median (IQR) | 32.0 (27.0, 38.0) | 33.5 (26.0, 41.0) | 0.31 |
| Static compliance before supine, median (IQR) | 32.8 (24.0, 42.2) | 33.0 (25.5, 40.0) | 0.85 |
| Static compliance after supine, median (IQR) | 32.1 (25.7, 40.0) | 32.7 (25.6, 40.0) | 0.74 |
| PaO_2_:FiO_2_ ratio, at day 2 in prone positioning, median (IQR) | 204.6 (163.6, 253.4) | 201.4 (157.4, 261.4) | 0.92 |
| PaO_2_:FiO_2_ ratio before supine, median (IQR) | 230.0 (175.0, 295.2) | 228.6 (189.8, 273.7) | 0.79 |
| PaO_2_:FiO_2_ ratio after supine, median (IQR) | 216.3 (165.0, 258.7) | 209.5 (166.1, 250.0) | 0.64 |
| Ventilatory ratio at day 2 in prone position, median (IQR) | 1.9 (1.6, 2.2) | 2.0 (1.7, 2.4) | 0.089 |
| Ventilatory ratio before supine, median (IQR) | 1.8 (1.5, 2.2) | 1.9 (1.7, 2.3) | 0.12 |
| Ventilatory ratio after supine, median (IQR) | 1.8 (1.5, 2.1) | 1.8 (1.5, 2.2) | 0.42 |

Main analysis is the Cox model for 90-days survival including as independent variables groups of prone positioning, SOFA Score, vasoactive support (first day in PP), and respiratory variables at day 2 (ventilatory ratio, PaO_2_:FiO_2_ ratio, static Compliance).

**Table S5. Respiratory and hemodynamic parameters before and after starting invasive mechanical ventilation according to duration of the first prone session**

|  | **Total**  **(n=417)** | **Group A (n=191)** | **Group B (n=128)** | **Group C (n=98)** | **p-value** |
| --- | --- | --- | --- | --- | --- |
| ***Before intubation*** | | | | | |
| Ventilatory support, n (%) |  |  |  |  | 0.002 |
| Usual nasal cannula | 13 (3.5) | 9 (5.2) | 2 (1.8) | 2 (2.3) |  |
| Venturi mask | 21 (5.7) | 9 (5.2) | 10 (9) | 2 (2.3) |  |
| Non-Rebreather mask | 122 (32.8) | 65 (37.6) | 34 (30.6) | 23 (26.1) |  |
| High flow nasal cannula | 180 (48.4) | 66 (38.2) | 55 (49.5) | 59 (67) |  |
| Non-invasive ventilation | 35 (9.4) | 23 (13.3) | 10 (9) | 2 (2.3) |  |
| Respiratory rate, breaths per minute, median (IQR) | 30 (26-35) | 30 (26-36) | 30 (25-35) | 30 (27-35) | 0.756 |
| PaO_2_ mm Hg, median (IQR) | 66 (57-78) | 67 (58-78) | 67 (57-81) | 61 (56-72) | 0.032 |
| FiO_2_ %, median (IQR) | 80 (60-95) | 80 (50-95) | 80 (70-100) | 80 (70-95) | 0.210 |
| PaO_2_:FiO_2_ ratio, median (IQR) | 86 (67-128) | 98 (72-150) | 86 (72-110) | 77 (64-100) | <0.001 |
| PaCO_2_, mm Hg, median (IQR) | 35 (32-40) | 35 (31-39) | 35 (32-41) | 35 (32-40) | 0.348 |
| pH, median (IQR) | 7.42 (7.38-7.46) | 7.42 (7.38-7.46) | 7.42 (7.37-7.46) | 7.42 (7.36-7.45) | 0.796 |
| Mean arterial pressure, mm Hg, median (IQR) | 87 (80-97) | 89 (80-100) | 88 (79-97) | 85 (79-95) | 0.167 |
| Heart rat, beats per minute, median (IQR) | 93 (79-104) | 91 (75-104) | 96 (80-103) | 95 (85-104) | 0.159 |
| Vasoactive support, n (%) | 25 (8.4) | 11 (8.7) | 12 (12.4) | 2 (2.6) | 0.061 |
|  |  |  |  |  |  |
| ***After intubation*** | | | | | |
| Patient positioning, n (%) |  |  |  |  | 0.169 |
| Supine | 360 (91.6) | 158 (89.3) | 111 (91.7) | 91 (95.8) |  |
| Prone | 33 (8.4) | 19 (10.7) | 10 (8.3) | 4 (4.2) |  |
| Ventilatory mode, n (%) |  |  |  |  | 1.000 |
| Volume assist/control | 390 (98.2) | 176 (98.3) | 121 (98.4) | 93 (97.9) |  |
| Pressure assist/control | 7 (1.8) | 3 (1.7) | 2 (1.6) | 2 (2.1) |  |
| Neuromuscular blockage, median (IQR) | 262 (66.8) | 121 (68.4) | 79 (65.3) | 62 (66.0) | 0.828 |
| Respiratory rate, breaths per minute, median (IQR) | 25 (22-28) | 24 (22-28) | 25 (22-30) | 26 (23-28) | 0.026 |
| Tidal volume, mL/kg IBW, median (IQR) | 6.1 (5.7-6.7) | 6.1 (5.6-6.7) | 6.1 (5.7-6.9) | 6.1 (5.7-6.8) | 0.92 |
| Plateau pressure, cm H_2_O, median (IQR) | 24 (21-26) | 22 (20-25) | 24 (21-27) | 24 (22-27) | 0.001 |
| Driving pressure, cm H_2_O, median (IQR) | 12 (11-15) | 12 (10-14) | 13 (11-15) | 13 (12-15) | 0.008 |
| PEEP, cm H_2_O, median (IQR) | 10 (8-12) | 10 (8-12) | 10 (8-14) | 10 (9-12) | 0.334 |
| Static compliance, mL/cm H_2_O, median (IQR) | 31 (26-37) | 32 (26-38) | 30 (26-35) | 30 (25-35) | 0.308 |
| PaO_2_, mm Hg, median (IQR) | 80 (69-96) | 82 (71- 99) | 79 (67-95) | 77 (66-91) | 0.173 |
| FiO_2_ %, median (IQR) | 0.7 (0.5-1.0) | 0.7 (0.5-1.0) | 0.7 (05-100) | 80 (60-100) | 0.087 |
| PaO_2_:FiO_2_ ratio, median (IQR) | 121 (85-167) | 127 (92-175) | 123 (83-177) | 108 (81-142) | 0.007 |
| PaCO_2_ mm Hg, median (IQR) | 46 (39-54) | 44 (38-50) | 46 (37-55) | 49 (42-57) | <0.001 |
| pH, median (IQR) | 7.31 (7.24-7.38) | 7.33 (7.26-7.39) | 7.32 (7.23-7.38) | 7.29 (7.22-7.34) | 0.004 |
| Ventilatory ratio, median (IQR) | 1.86 (1.51-2.40) | 1.71 (1.44-2.21) | 1.95 (1.50-2.44) | 2.14 (1.62-2.57) | <0.001 |
| Mean arterial pressure, mm Hg | 78 (70-88) | 79 (70-90) | 79 (71-88) | 74 (70-87) | 0.421 |
| Heart rate, beats per minute, median (IQR) | 85 (73-100) | 81 (71-97) | 87 (78-100) | 91 (79-105) | 0.004 |
| Vasoactive support, n (%) | 256 (71.7) | 110 (70.1) | 83 (72.8) | 63 (73.3) | 0.857 |

Group A patients remained 2-to-3 days in prone position during their first session. Group B patients remained 4-to-5 days in prone position during their first session. Group C patients remained more than 5 days in prone position during their first session. The data reported as Before starting invasive mechanical ventilation (upper half of the table) corresponds to the last data registered in the clinical files before intubation, while the data reported as After starting invasive mechanical ventilation (lower half of the table) corresponds to the first data registered in the clinical files collected the patient was intubated and connected to mechanical ventilation. IBW: ideal body weight, PEEP: positive end-expiratory pressure, PaO_2_: partial pressure of arterial oxygen, FiO_2_: Inspired oxygen fraction, PaO_2_:FiO_2_ ratio: ratio of partial pressure of arterial oxygen to inspired oxygen fraction, PaCO_2_: partial pressure of arterial carbon dioxide, PEEP: Positive end-expiratory pressure, Static compliance: Static respiratory system compliance, Ventilatory ratio is a unit less index calculated as (minute ventilation in mL/min x PaCO_2_) / (Ideal body weight x 100 x 37.5).

**Table S6. Sedative agents, Opioids and Neuromuscular Blockade before and after prone position initiation**

| **Sedatives/Opioids/NMB** | **Group A** | |  | **Group B** | |  | **Group C** | |  |
| --- | --- | --- | --- | --- | --- | --- | --- | --- | --- |
|  |  |  |  |  |  |  |  |  |  |
|  | Before PP | PP day 1 | p-value | Before PP | PP day 1 | p-value | Before PP | PP day 1 | p-value |
|  |  |  |  |  |  |  |  |  |  |
| Propofol | 28 (16.0%) | 28 (15.7%) | 1.000 | 18 (15.5%) | 14 (12.1%) | 0.568 | 36 (40%) | 32 (34%) | 0.445 |
| Midazolam | 125 (71.4%) | 131 (73.6%) | 0.721 | 94 (81.0%) | 93 (80.2%) | 1.000 | 53 (60%) | 60 (65%) | 0.542 |
| Propofol + Midazolam | 16 (9.1%) | 14 (7.9%) | 0.706 | 4 (3.4%) | 6 (5.2%) | 0.748 | 0 (0%) | 1 (1%) | 1.000 |
| Other | 6 (3.4%) | 5 (2.8%) | 0.769 | 0 (0%) | 3 (2.6%) | 0.247 | 0 (0%) | 0 (0%) | - |
| Fentanyl | 174 (91.1%) | 179 (93.7%) | 0.442 | 115 (89.8%) | 119 (93.0%) | 0.504 | 88 (89.8%) | 93 (94.9%) | 0.282 |
| NMB | 151 (81.6%) | 182 (95.8%) | <0.001 | 99 (80.5%) | 122 (96.1%) | <0.001 | 78 (81.2%) | 97 (100%) | <0.001 |
|  |  |  |  |  |  |  |  |  |  |
| **Dose** |  |  |  |  |  |  |  |  |  |
| Propofol (mg/Kg/h), mean (SD) | 2.7 ± 1.3 | 2.6 ± 1.4 | 0.797 | 2.7 ± 1.0 | 2.8 ± 1.3 | 0.916 | 2.7 ± 0.7 | 2.7 ± 0.8 | 0.149 |
| Midazolam (mg/Kg/h), mean (SD) | 0.075 ± 0.03 | 0.080 ± 0.03 | 0.029 | 0.091 ± 0.03 | 0.097 ± 0.03 | 0.117 | 0.096 ± 0.03 | 0.098 ± 0.03 | 0.278 |
| Fentanyl (mcg/Kg/h), mean (SD) | 3.0 ± 1.0 | 3.1 ± 1.0 | 0.022 | 3.2 ± 1.0 | 3.4 ± 1.0 | 0.003 | 3.1 ± 0.7 | 3.2 ± 0.8 | 0.131 |

**Table S7. Description of prone positioning sessions according to the number of sessions required**

| **Number of prone sessions required** | **Frequency**  **n (%)** | **Session length**  **days, median (IQR)** | | | | **Cumulative days in prone**  **days**, **median (IQR)** | **** Relative time in prone**  **%, median (IQR)** |
| --- | --- | --- | --- | --- | --- | --- | --- |
|  |  | **First** | **Second** | **Third** | **Fourth** |  |  |
| One | 318 (76.3) | 4 (3-6) | - | - | - | 4 (3-6) | 100 (100-100) |
| Two | 88 (21.1) | 3 (2-5) | 4 (3-5) | - | - | 7 (6-10) | 80 (60-88) |
| Three | 9 (2.1) | 4 (2-5) | 4 (3-4) | 3 (3-5) | - | 11 (9-18) | 67 (62-73) |
| Four | 2 (0.5) | 2 and 5* | 1 and 3* | 1 and 6* | 3 and 4* | 9 and 16* | 67 and 75* |
| Total cohort | 417 (100) | 4 (3-5) | 4 (3-5) | 3 (3-6) | 3 and 4* | 4 (3-7) | 100 (100-100) |

Data are n (%) or median (IQR), except for data corresponding to only two patients in which case the individual data are presented (*).
**: Cumulative days on prone position / Total days elapsed between the start of the first session and the end of the last session.

**Table S8. Reasons for interrupting prone sessions not related to oxygenation improvement**

|  | **First prone session**  **n (%)** | **Cumulative incidence***  **n (%)** |
| --- | --- | --- |
| Cardiac arrest | 3 (0.7) | 5 (1.2) |
| Heart rate < 30 beats/min for more than 1 minute | - | - |
| Hemoptysis | - | 2 (0.5) |
| PaO_2_:FIO_2_ ratio deterioration by more than 25 % | 14 (3.4) | 17 (4.1) |
| SpO_2_ < 85% or PaO_2_ < 55 mm Hg for more than 5 minutes under FIO_2_ 1 | 5 (1.2) | 6 (1.4) |
| Any other life-threatening reason for which the clinician decided to stop | 25 (6) | 32 (7.7) |
| Any other clinical decision for which attending decided the change to supine | 66 (15.8) | - |

* Cumulative incidence considering all prone position sessions.

**Table S9. Variables associated to group classification according to the duration of the first prone session**

| **Variable** | **Odds ratio (CI 95%)** | **p-value** |
| --- | --- | --- |
| ***Basal characteristics*** | | |
| Ideal body weight | 1.031 (1.001–1.061) | 0.039 |
| Coronary heart disease | 2.305 (0.892–5.957) | 0.085 |
| Sepsis | 1.772 (1.162–2.704) | 0.008 |
| SOFA score | 0.931 (0.862–1.005) | 0.066 |
| Days from mechanical ventilation to prone position | 0.962 (0.919–1.006) | 0.089 |
| ***Before Intubation*** | | |
| Mean arterial pressure, mmHg | 0.987 (0.974–1.000) | 0.050 |
| PaO_2_:FiO_2_ ratio | 0.992 (0.988–0.996) | <0.001 |
| FiO_2_ | 1.013 (1.003–1.022) | 0.008 |
| ***After Intubation*** | | |
| PaO_2_:FiO_2_ ratio | 0.997 (0.994–0.999) | 0.014 |
| FiO_2_ | 1.008 (1.000–1.016) | 0.057 |
| PaCO_2_ | 1.020 (1.007–1.034) | 0.004 |
| pH | 0.070 (0.011–0.461) | 0.006 |
| Ventilatory ratio | 1.544 (1.199–1.988) | 0.001 |
| Static compliance | 1.100 (1.026–0.179) | 0.007 |
| ***Before starting prone positioning*** | | |
| PaO_2_:FiO_2_ ratio | 0.994 (0.990–0.997) | 0.002 |
| FiO_2_ | 1.014 (1.005–1.022) | 0.001 |
| PaCO_2_, | 1.023 (1.009–1.036) | 0.001 |
| Ventilatory ratio | 1.665 (1.282–2.161) | <0.001 |
| ***At day 1 in prone positioning*** | | |
| PaO_2_:FiO_2_ ratio | 0.991 (0.988–0.994) | <0.001 |
| FiO_2_, % | 1.039 (1.027–1.051) | <0.001 |
| PaCO_2_, | 1.039 (1.020–1.058) | <0.001 |
| pH | 0.054 (0.007–0.444) | 0.007 |
| Ventilatory ratio | 2.371 (1.727–3.255) | <0.001 |
| Static compliance | 0.971 (0.952–0.989) | 0.002 |
| Driving pressure | 1.123 (1.046–1.205) | 0.001 |
| ***At day 2 in prone positioning*** | | |
| Respiratory rate, breath per minute | 1.069 (1.030–1.109) | <0.001 |
| PaO_2_:FiO_2_ | 0.987 (0.984–0.990) | <0.001 |
| FiO_2_ | 1.065 (1.049–1.081) | <0.001 |
| PaCO_2_ | 1.034 (1.015–1.052) | <0.001 |
| pH | 0.941 (0.010–0.886) | 0.039 |
| Ventilatory ratio | 2.065 (1.524–2.799) | <0.001 |
| Plateau pressure | 1.120 (1.063–1.181) | <0.001 |
| Driving pressure | 1.150 (1.081–1.222) | <0.001 |
| Static compliance | 0.966 (0.948–0.985) | <0.001 |

SOFA, sequential organ failure assessment; FiO_2_: Inspired oxygen fraction, PaO_2_: partial pressure of arterial oxygen; PaO_2_:FiO_2_ ratio: ratio of partial pressure of arterial oxygen to inspired oxygen fraction, PaCO_2_: partial pressure of arterial carbon dioxide, Ventilatory ratio is a unit less index calculated as (minute ventilation in ml/min x PaCO_2_) / (predicted bodyweight x 100 x 37.5).

**Table S10. Risks factors for 90-days mortality**

| **Variable** | **Univariate HR (CI 95%)** | **p-value** |
| --- | --- | --- |
| ***Basal characteristics*** | | |
| Age, years | 1.052 (1.036 – 1.068) | <0.001 |
| Hypertension | 1.925 (1.366 – 2.711) | <0.001 |
| T2 Diabetes Mellitus | 1.366 (0.992 – 1.881) | 0.056 |
| Immunosuppression | 2.991 (1.224 – 7.307) | 0.016 |
| Chronic Kidney Disease | 3.578 (2.096 – 6.108) | <0.001 |
| SOFA score | 1.126 (1.060 – 1.195) | <0.001 |
| APACHE score | 1.050 (1.027 – 1.073) | <0.001 |
| ***Before Intubation*** | | |
| Mean arterial pressure | 0.985 (0.973 – 0.998) | 0.023 |
| Vasoactive support | 1.797 (1.009 – 3.202) | 0.047 |
| pH | 0.148 (0.023 – 0.936) | 0.042 |
| ***After Intubation*** | | |
| PaCO_2_ | 1.012 (1.002 – 1.023) | 0.021 |
| pH | 0.033 (0.007 – 0.156) | <0.001 |
| ***Before starting prone positioning*** | | |
| Vasoactive support | 1.489 (1.010 – 2.196) | 0.044 |
| PaO_2_:FiO_2_ ratio | 0.996 (0.993 – 1.000) | 0.027 |
| FiO_2_ | 1.007 (1.00 – 1.015) | 0.054 |
| PaCO_2_ | 1.016 (1.006 – 1.027) | 0.002 |
| pH | 0.028 (0.006 – 0.120) | <0.001 |
| Ventilatory ratio | 1.335 (1.074 – 1.659) | 0.009 |
| ***At day 1 in prone positioning*** | | |
| Vasoactive support | 1.557 (1.020 – 2.378) | 0.040 |
| Heart rate | 1.009 (1.001 – 1.018) | 0.037 |
| FiO_2_ | 1.017 (1.008 – 1.026) | <0.001 |
| PaCO_2_ | 1.034 (1.019 – 1.051) | <0.001 |
| pH | 0.008 (0.001 – 0.047) | <0.001 |
| Ventilatory ratio | 1.663 (1.287 – 2.150) | <0.000 |
| Static compliance | 0.979 (0.962 – 0.996) | 0.014 |
| Driving pressure | 1.076 (1.014 – 1.141) | 0.015 |
| ***At day 2 in prone positioning*** | | |
| Respiratory rate | 1.035 (1.002 – 1.068) | 0.036 |
| PaO_2_:FiO_2_ ratio | 0.995 (0.993 – 0.997) | <0.001 |
| FiO_2_ | 1.022 (1.013 – 1.032) | <0.001 |
| PaCO_2_ | 1.035 (1.021 – 1.050) | <0.001 |
| pH | 0.001 (0.000 – 0.008) | <0.001 |
| Ventilatory ratio | 1.656 (1.318 – 2.081) | <0.001 |
| Plateau pressure | 1.054 (1.008 – 1.103) | 0.022 |
| Driving pressure | 1.071 (1.024 – 1.121) | 0.003 |
| Static compliance | 0.980 (0.964 – 0.997) | 0.023 |
| Prone session duration | 1.075 (1.021 – 1.132) | 0.006 |

SOFA, sequential organ failure assessment; APACHE, acute physiology and chronic health evaluation. FiO_2_: Inspired oxygen fraction, PaO_2_: partial pressure of arterial oxygen; PaO_2_:FiO_2_ ratio: ratio of partial pressure of arterial oxygen to inspired oxygen fraction, PaCO_2_: partial pressure of arterial carbon dioxide, Ventilatory ratio is a unitless index calculated as (minute ventilation in ml/min x PaCO_2_) / (predicted bodyweight x 100 x 37.5).

**Table S11. Large cohort studies of Covid-19 patients treated with mechanical ventilation and prone positioning**

| **Study** | **Patients** | **Age** | **SOFA** | **APACHE II** | **PaO_2_:FiO_2_** | ***Session duration** | **Sessions** | **Time on MV** | **Survival** | | |
| --- | --- | --- | --- | --- | --- | --- | --- | --- | --- | --- | --- |
|  | **(n)** | **(y.o.)** |  |  | **(mm Hg)** | **(hours/days)** | **(n)** | **(days)** | **Hospital, (%)** | **ICU, (%)** | **90-day, (%)** |
| Camporota 2021(2) | 220 | 62 (56-71) | 6 (4-8) |  | 98 (78-120) | 16 (15.5-18) hours | 4 (2-6) | NR | 57 | 57 | NR |
| Langer 2021(3) | 648 | 63 (55-69) | 4 (3-5) | 10 (8-13) | 98 (72–121) | 18.5 (16-22) hours | 3 (1-4) | 16 (10-30) | 55 | 59 | NR |
| Stilma 2021(4) | 438 | 63.8 (10.8) | 8.6 (4.1) | 19.3 (8.9) | 120 (90-150) | 15 (10.5-21) hours | 3 (2-3) | NR | NR | NR | 61.4 |
| Scaramuzzo 2021(5) | 191 | 66 (59-72) | 5 (3-7) |  | 103 (85-128) | 16 (16–17) hours | NR | 18 (11–28) | NR | 56.6 | NR |
| Estenssoro 2021(6) | 1176 | 62 (52-70) | 5 (3-8) | 15 (10-20) | NR | 24 (21-36) hours | 2 (2-4) | NR | 36.5 | NR | NR |
| REVA Network 2022(7) | 1504 | 63 (54-71) | 5 (3-8) |  | 128 (88-178)** | 20 (16-32) hours | 4 (2-6) | NR | NR | NR | 60 |
| Patel 2022(8) | 270 | 59 (51-66) | 9 (7-11) |  | 123 (91-164) | 2 (1-4) days | 1 (1-2) | 16 (10-26) | NR | 47.4% | NR |
| Cornejo 2022 | 417 | 62 (52-68) | 5 (4-7) | 14 (10-18) | 119 (85-154) | 4 (3-5) days | 1 (1-1) | 15 (10--22) | 66.7 | 68.6 | 63.8 |

* Time in hours or days, ** PaO_2_:FiO_2_ ratio was only available for the early prone subgroup, MV: Mechanical ventilation, NR: Not reported.

Variables are reported as median [IQR] or absolute frequency and percentages

**Table S12. Studies of prolonged prone positioning**

| **Study** | **Diagnosis** | **Design** | **Patients**  **(n)** | **Session duration**  * **(hours/days)** | **Sessions**  **(n)** | **Pressure sores**  **(%)** | **Survival**  **(%**)** |
| --- | --- | --- | --- | --- | --- | --- | --- |
| Chan 2007(9) | ARDS | S-Prospective | 11 | > 72 hours | 1 (1-1) | 18.2% | 63.6** |
| Romero 2009(10) | ARDS | S-Prospective | 15 | 55±7 hours | 1 (1-1) | 13.3% | 60 |
| Lee K 2010(11) | ARDS | S-Retrospective | 96 | 79±61 hours | NR | 20% | 44** |
| Cornejo 2011(12) | H1N1 | S-Retrospective | 10 | 82±49 hours | 1 (1-2) | 50% | 80 |
| Hernandez 2019(13) | ARDS | S-Retrospective | 7 | 57±17 hours | 1 (1-1) | 57.1% | 100** |
| Carsetti 2020(14) | COVID-19 | S-Retrospective | 10 | 36 (33-39) hours | NR | NR | NR |
| Rezoagli 2021(15) | COVID-19 | S-Retrospective | 15 | 39±6 hours | 2 (2-4) | 67% | 67** |
| Parker 2021(16) | COVID-19 | S-Retrospective | 12 | 57 (45-66) hours | NR | NR | 67 |
| Douglas 2021(17) | COVID-19 | S-Retrospective | 61 | 3 (2-5) days | 1 (1-1.5) | 65.6% | 68.9 |
| Lee P 2021(18) | ARDS | S-Retrospective | 116 | 66 (44-85) hours | NR | NR | 44.8** |
| Lucchini 2021(19) | COVID-19 | S-Retrospective | 37 | 34 (30-41) hours | 3 (2-4) | 51% | 84** |
| Concha 2022(20) | COVID-19 | S-Retrospective | 17 | 48±18 hours | 3±1 | 52.9% | 82 |
| Garg 2022(21) | COVID-19 | S-Retrospective | 10 | 60 (66-71) hours | 2 (2-2) | 10% | 100 |
| Cornejo 2022 | COVID-19 | M-Retrospective | 417 | 4 (3-5) days | 1 (1-1) | 36.2% | 66.7 |

* Time in hours or days, and in median (IQR) or mean ± SD, according to the data provided in the original publication.

** This study did not report hospital survival, so ICU survival is shown.

S: Single-centre, M: Multicentre, NR: Not reported

**Figure S1. Algorithm for the management of COVID-19 patients with Acute Respiratory Failure (Chilean Society of Intensive Care Medicine)**


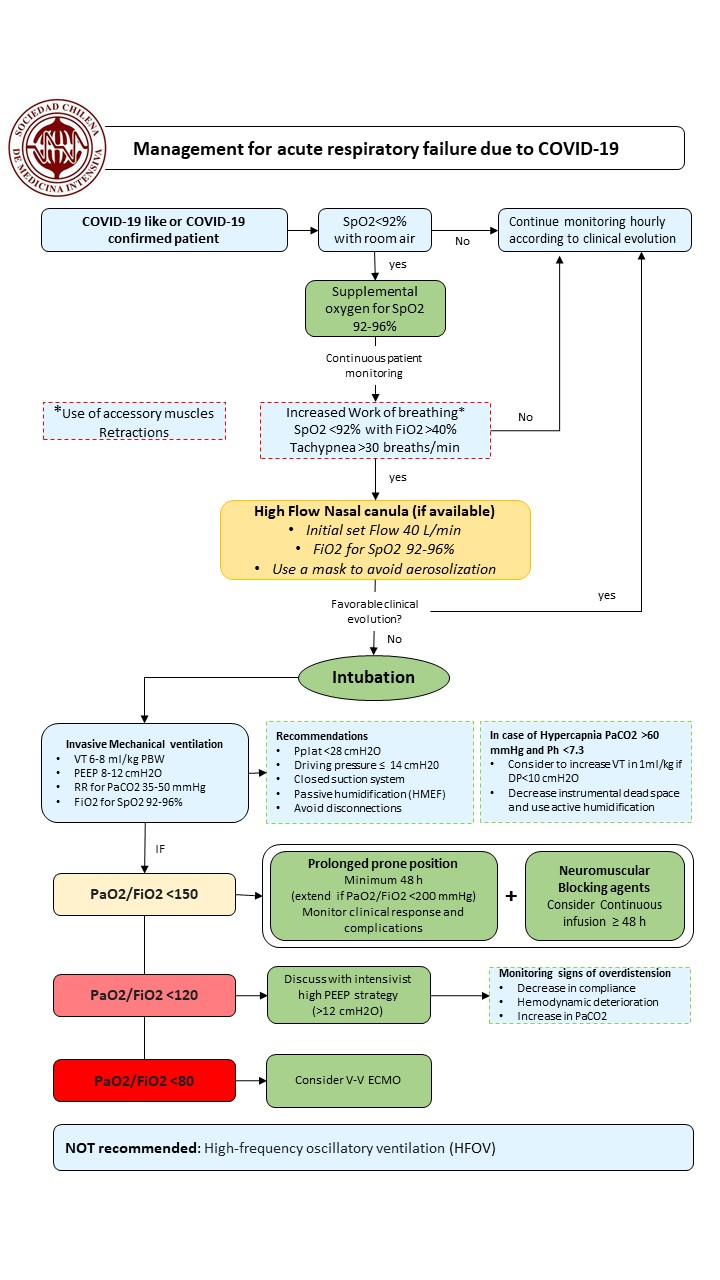


# Figure S2. Cohort flowchart for patients treated with prolonged prone positioning

Patients treated with prolonged prone positioning recruited in the 8-weeks enrolment window

(n = 547)

Patients excluded (n=130)

Causes of exclusion:

- 20 patients presented prone sessions lower than 48 continuous hours
- 4 patients had duplicated registers
- 106 patients were excluded due to significant missing data

Enrollment

#

Patients treated with

prolonged prone positioning

finally included

(n=417)

**Follow up**

Patients treated with

prolonged prone positioning followed until day 90

(n=417)

Analysis

Patients treated with

Prolonged prone positioning

finally analyzed

(n=417)

**Figure S3. Distribution of patients according to the first prone session duration (days)**

**
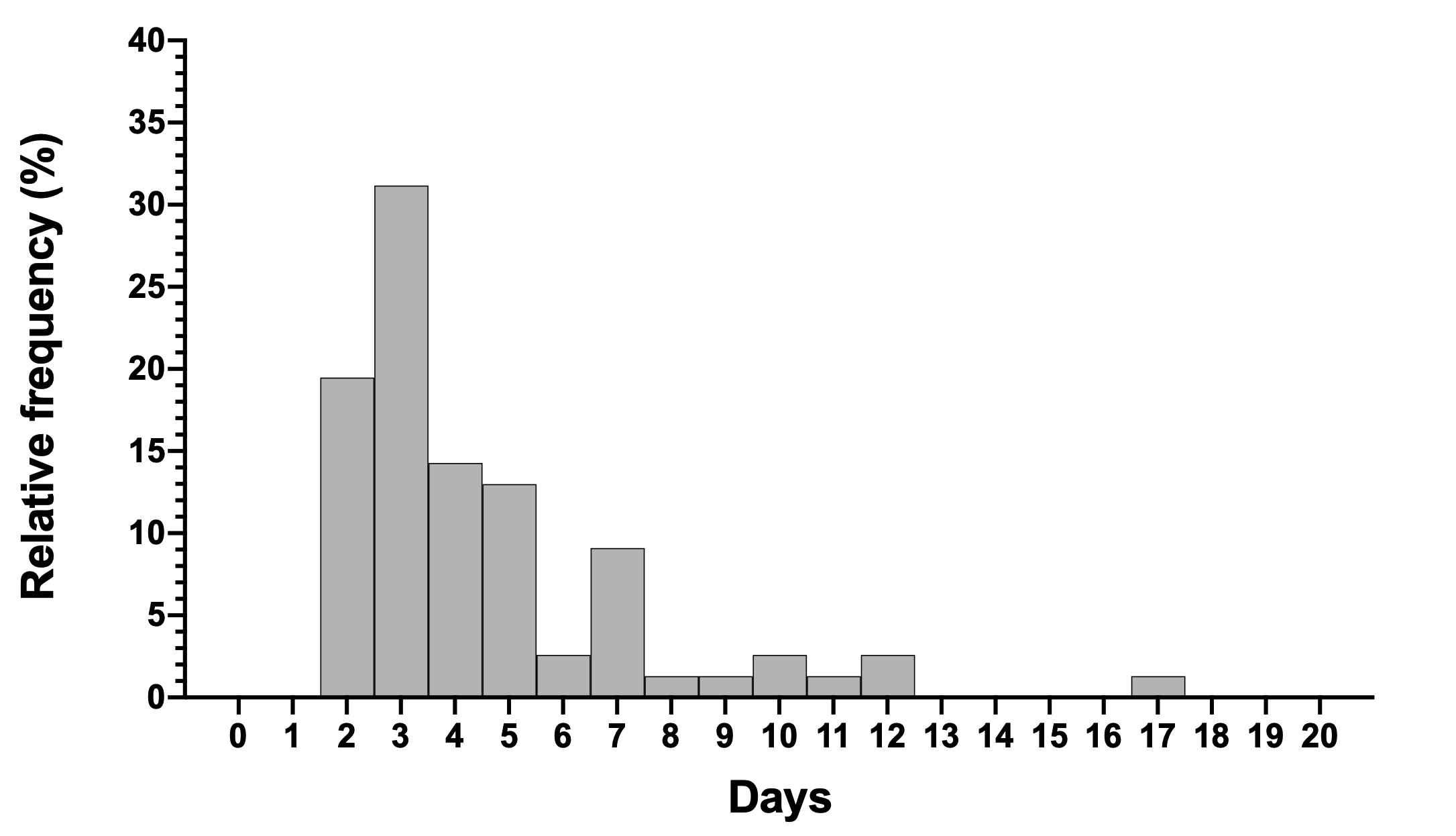
**

**Figure S4.** Correlation between PaO_2_:FiO_2_ ratio in prone before supine and PaO_2_:FiO_2_ ratio in supine after prone

Red dotted lines represent the oxygenation thresholds in prone to turn the patients to supine, and in supine to turn the patients to prone

**Figure S5: Directed acyclic graph to select the confounding factors**

**
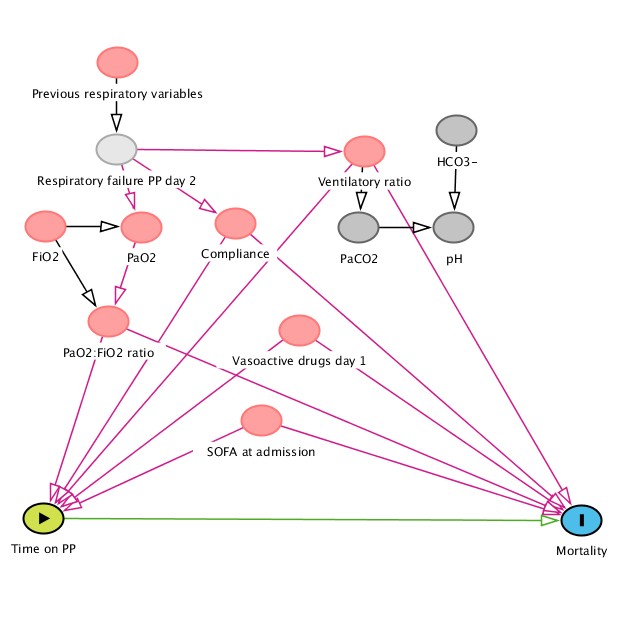
**

PP: Prone positioning; PaO_2_: partial pressure of arterial oxygen; PaO_2_:FiO_2_ ratio: ratio of partial pressure of arterial oxygen to inspired oxygen fraction; Ventilatory ratio is a unitless index calculated as (minute ventilation in ml/min x PaCO_2_) / (predicted bodyweight x 100 x 37.5). PaCO_2_: partial pressure of arterial carbon dioxide; SOFA, sequential organ failure assessment; Vasoactive: Vasoactive support

**References**

1. Diaz RA, Graf J, Zambrano JM, Ruiz C, Espinoza JA, Bravo SI, Salazar PA, Bahamondes JC, Castillo LB, Gajardo AIJ, Kursbaum A, Ferreira LL, Valenzuela J, Castillo RE, Pérez-Araos RA, Bravo M, Aquevedo AF, González MG, Pereira R, Ortega L, Santis C, Fernández PA, Cortés V, Cornejo RA. Extracorporeal Membrane Oxygenation for COVID-19-associated Severe Acute Respiratory Distress Syndrome in Chile A Nationwide Incidence and Cohort Study. *Am J Respir Crit Care Med* 2021;204:34–43.

2. Camporota L, Sanderson B, Chiumello D, Terzi N, Argaud L, Rimmelé T, Metuor R, Verstraete A, Cour M, Bohé J, Piriou V, Beuret P, Guérin C. Prone Position in Coronavirus Disease 2019 and Noncoronavirus Disease 2019 Acute Respiratory Distress Syndrome. *Crit Care Med* 2021;Publish Ah:

3. Langer T, Brioni M, Guzzardella A, Carlesso E, Cabrini L, Castelli G, Dalla Corte F, De Robertis E, Favarato M, Forastieri A, Forlini C, Girardis M, Grieco DL, Mirabella L, Noseda V, Previtali P, Protti A, Rona R, Tardini F, Tonetti T, Zannoni F, Antonelli M, Foti G, Ranieri M, Pesenti A, Fumagalli R, Grasselli G, Berselli A, Bove T, *et al.* Prone position in intubated, mechanically ventilated patients with COVID-19: a multi-centric study of more than 1000 patients. *Crit Care* 2021;25:1–11.

4. Stilma W, Van Meenen DMP, Valk CMA, De Bruin H, Paulus F, Neto AS, Schultz MJ. Incidence and practice of early prone positioning in invasively ventilated COVID-19 patients—insights from the PRoVENTCOVID observational study. *J Clin Med* 2021;10:4783.

5. Scaramuzzo G, Gamberini L, Tonetti T, Zani G, Ottaviani I, Mazzoli CA, Capozzi C, Giampalma E, Letizia M, Reggiani B, Bertellini E, Castelli A, Cavalli I, Colombo D, Crimaldi F, Damiani F, Fusari M, Gamberini E, Gordini G, Laici C, Lanza MC, Leo M, Marudi A, Nardi G, Papa R, Potalivo A, Russo E, Taddei S, Consales G, *et al.* Sustained oxygenation improvement after first prone positioning is associated with liberation from mechanical ventilation and mortality in critically ill COVID-19 patients: a cohort study. *Ann Intensive Care* 2021;11:63.

6. Estenssoro E, Loudet CI, Ríos FG, Kanoore Edul VS, Plotnikow G, Andrian M, Romero I, Piezny D, Bezzi M, Mandich V, Groer C, Torres S, Orlandi C, Rubatto Birri PN, Valenti MF, Cunto E, Sáenz MG, Tiribelli N, Aphalo V, Reina R, Dubin A, Estenssoro E, Dubin A, Loudet CI, Ríos F, Kanoore Edul VS, Plotnikow G, Reina R, Andrian M, *et al.* Clinical characteristics and outcomes of invasively ventilated patients with COVID-19 in Argentina (SATICOVID): a prospective, multicentre cohort study. *Lancet Respir Med* 2021;9:989–998.

7. Le Terrier C, Sigaud F, Lebbah S, Desmedt L, Hajage D, Guérin C, Pugin J, Primmaz S, Terzi N, Mercat A, Asfar P, Beloncle F, Demiselle J, Pham T, Pavot A, Monnet X, Richard C, Demoule A, Dres M, Mayaux J, Beurton A, Daubin C, Descamps R, Joret A, Du Cheyron D, Pene F, Chiche J-D, Jozwiak M, Jaubert P, *et al.* Early prone positioning in acute respiratory distress syndrome related to COVID-19: a propensity score analysis from the multicentric cohort COVID-ICU network—the ProneCOVID study. *Crit Care* 2022;26:1–14.

8. Patel B V., Haar S, Handslip R, Auepanwiriyakul C, Lee TML, Patel S, Harston JA, Hosking-Jervis F, Kelly D, Sanderson B, Borgatta B, Tatham K, Welters I, Camporota L, Gordon AC, Komorowski M, Antcliffe D, Prowle JR, Puthucheary Z, Faisal AA, Patel B, Addie E, Chisholm R, Crocokft A, Gilfedder A, Harding P, Madzamba G, Mathai N, Patel M, *et al.* Natural history, trajectory, and management of mechanically ventilated COVID-19 patients in the United Kingdom. *Intensive Care Med* 2021;47:549–565.

9. Chan M-C, Hsu J-Y, Liu H-H, Lee Y-L, Pong S-C, Chang L-Y, Kuo BI-T, Wu C-L. Effects of Prone Position on Inflammatory Markers in Patients with ARDS Due to Community-acquired Pneumonia. *J Formos Med Assoc* 2007;106:708–716.

10. Romero CM, Cornejo RA, Gálvez LR, Llanos OP, Tobar EA, Berasaín MA, Arellano DH, Larrondo JF, Castro JS. Extended prone position ventilation in severe acute respiratory distress syndrome: A pilot feasibility study. *J Crit Care* 2009;24:81–88.

11. Lee K, Kim MY, Yoo JW, Hong SB, Lim CM, Koh Y. Clinical meaning of early oxygenation improvement in severe acute respiratory distress syndrome under prolonged prone positioning. *Korean J Intern Med* 2010;25:58–65.

12. Cornejo R, Tobar E, Díaz G, Romero C, Llanos O, Gálvez LR, Zamorano A, Fábrega L, Neira W, Arellano D, Repetto C, Aedo D, Díaz JC, González R. Systematic approach for severe respiratory failure due to novel a (H1N1) influenza. *Minerva Anestesiol* 2011;77:510–521.

13. Hernández G, Gorordo L, Hernández M, Zamora S, Carrasco M, Toledo M. Ventilación en posición prono en pacientes postoperados de cirugía abdominal complicados con síndrome de dificultad respiratoria aguda: análisis de una cohorte. *Med Crítica* 2019;33:245–250.

14. Carsetti A, Paciarini AD, Marini B, Pantanetti S, Adrario E, Donati A. Prolonged prone position ventilation for SARS-CoV-2 patients is feasible and effective. *Crit Care* 2020;24:1–3.

15. Rezoagli E, Mariani I, Rona R, Foti G, Bellani G. Difference between prolonged versus standard duration of prone position in COVID-19 patients: a retrospective study. *Minerva Anestesiol* 2021;87:1383–1385.

16. Parker EM, Bittner EA, Berra L, Pino RM. Efficiency of prolonged prone positioning for mechanically ventilated patients infected with covid-19. *J Clin Med* 2021;10:.

17. Douglas IS, Rosenthal CA, Swanson DD, Hiller T, Oakes J, Bach J, Whelchel C, Pickering J, George T, Kearns M, Hanley M, Mould K, Roark S, Mansoori J, Mehta A, Schmidt EP, Neumeier A. Safety and Outcomes of Prolonged Usual Care Prone Position Mechanical Ventilation to Treat Acute Coronavirus Disease 2019 Hypoxemic Respiratory Failure∗. *Crit Care Med* 2021;490–502.doi:10.1097/CCM.0000000000004818.

18. Lee PH, Kuo CT, Hsu CY, Lin SP, Fu PK. Prognostic factors to predict icu mortality in patients with severe ards who received early and prolonged prone positioning therapy. *J Clin Med* 2021;10:.

19. Lucchini A, Russotto V, Barreca N, Villa M, Casartelli G, Marcolin Y, Zyberi B, Cavagnuolo D, Verzella G, Rona R, Fumagalli R, Foti G. Short and long-term complications due to standard and extended prone position cycles in CoViD-19 patients. *Intensive Crit Care Nurs* 2022;69:103158.

20. Concha P, Treso-Geira M, Esteve-Sala C, Prades-Berengué C, Domingo-Marco J, Roche-Campo F. Invasive mechanical ventilation and prolonged prone position during the COVID-19 pandemic. *Med intensiva* 2022;46:161–163.

21. Garg SK, Garg P. Safety of prolonged prone ventilation in critically ill COVID-19 patients: A short report. *J Infect Public Health* 2022;15:397–399.
